# Lithium Complexes Derived of Benzylphosphines: Synthesis, Characterization and Evaluation in the ROP of *rac*-Lactide and *ε*-Caprolactone

**DOI:** 10.3390/molecules23010082

**Published:** 2017-12-30

**Authors:** Ernesto Rufino-Felipe, Miguel-Ángel Muñoz-Hernández, Virginia Montiel-Palma

**Affiliations:** Centro de Investigaciones Químicas, Instituto de Investigación en Ciencias Básicas y Aplicadas, Universidad Autónoma del Estado de Morelos, Cuernavaca 62209, Mexico; erf@uaem.mx (E.R.-F.); mamund2@uaem.mx (M.-Á.M.-H.)

**Keywords:** organolithium, lithium organophosphines, DFT computations, ROP, *rac*-LA polymerization, *ε*-caprolactone polymerization

## Abstract

A series of lithium complexes ([Ph_2_P(*o*-C_6_H_4_-CH_2_Li·TMEDA)] (**1-Li**), [PhP(*o*-C_6_H_4_-CH_3_)(*o*-C_6_H_4_-CH_2_Li·TMEDA)] (**2-Li**), [PhP(*o*-C_6_H_4_-CH_2_Li·TMEDA)_2_] (**2-Li_2_**) and [P(*o*-C_6_H_4_-CH_2_Li·TMEDA)_3_] (**3-Li_3_**)) was prepared from mono-, di- and tri-benzylphosphines and varying amounts of *n*BuLi and was characterized extensively by IR and ^1^H, ^7^Li, ^13^C and ^31^P NMR spectroscopy. The molecular structures of complexes **1-Li** and **2-Li** were determined by single-crystal X-ray diffraction studies. The two complexes have monomeric structures in the solid state comprising seesaw lithium atoms. In each case, the ligand exhibits an asymmetric C-C η^2^-coordination mode and an intramolecular P-Li bond interaction. Theoretical calculations at Density functional theory (DFT) level M06/6111+G(2d,p) show that indeed a P-Li bond is established which can be explained as the P lone pair (*sp*^1.26^) being partially delocalized on an available *sp*^2^ orbital on Li (*sp*^2.04^) and additional bonding contribution of the phosphorous atom to Li stems from further delocalization of a σ P-C orbital into the *sp*^2^ orbital on Li. The observed short contact distances between an aromatic *ipso* carbon and Li in the crystal structures of **1-Li** and **2-Li** are explained as due to the interaction of a σ C-Li orbital into the π* orbital of a C-C aromatic bond. Preliminary tests show compounds **1-Li**, **2-Li**, **2-Li_2_** and **3-Li_3_** are active catalysts in the solvent free ring-opening polymerization (ROP) of *ε*-caprolactone (*ε*-CL) and *rac*-lactide (*rac*-LA). High conversions to polycaprolactones were obtained in short periods of time: 1–6 min at 25 °C. Additionally, all four lithium complexes behave as moderately good initiators for the ROP of *rac*-LA showing high conversions to polylactides at 140 °C in one hour.

## 1. Introduction

Modern life can no longer be conceived without high-value commercial polymers for a variety of applications in all aspects of human activity. Most of these polymers derive from hydrocarbons, in turn attained from non-renewable fossil fuels of limited availability. Hence, one of the biggest challenges for chemists is the development of new synthetic routes to obtain polymers and other compounds from renewable sources. In principle, there are two potentially promising renewable sources for ester-containing monomers: the first involves the use of atmospheric CO_2_, while the second uses cyclic esters as raw materials. Within the second category, the ring-opening polymerization (ROP) reactions of *rac*-lactide (LA) and *ε*-caprolactone (*ε*-CL), respectively, generate polylactides and polycaprolactones. Evidently, narrow distributions of their molecular weights are much desired. For these transformations, tin complexes have been used in industrial scale as catalysts, yet the toxicity of tin prevents the use of the resulting polymers in certain applications such as sutures or implants for biomedical applications [1,2,3]. An alternative option for these transformations involves the use of group 1 complexes which are reasonably effective, yet further developments are necessary to improve conversion yields and selectivities preferably in solvent free systems [4,5,6,7,8,9].

Independently, in our search for the synthesis of dual functionality silylphosphine ligands for transition metal complexation, we generate phosphines bearing organolithium groups as intermediates [10,11]; the chemical nature of which had not been previously investigated. Herein, we present our results on the bonding modes adopted by three benzylphosphines (**1**–**3**) coordinated to lithium. Four lithium complexes with one, two or three lithium atoms were isolated and spectroscopically characterized in solution and in the solid state. Theoretical calculations show excellent correlations with the experimental solid-state structures of two of these complexes and aid the understanding of the orbitals involved in the bonding and the unusual geometry exhibited by the metal. Eventually, the compounds were preliminarily tested in the ROP of cyclic esters *ε*-CL and *rac*-LA. We found these lithium phosphines are active catalysts in the solvent free ROP of *ε*-CL and *rac*-LA achieving high monomer conversions though low control of molecular weight distribution.

## 2. Results and Discussion

### 2.1. Synthesis and Characterization of Lithium Complexes Derived of Organophosphines

**Scheme 1:**
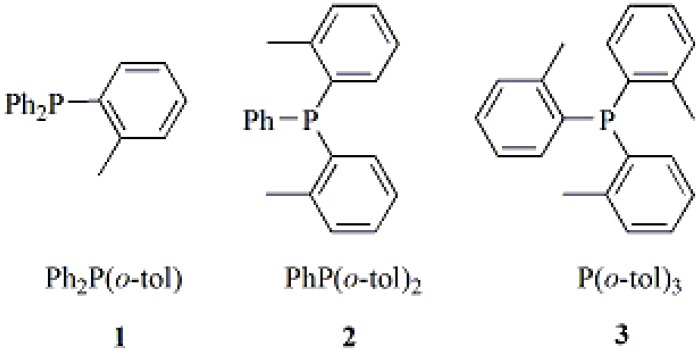
Structures of the benzylphosphines ligands **1**, **2** and **3**.

**Scheme 2:**
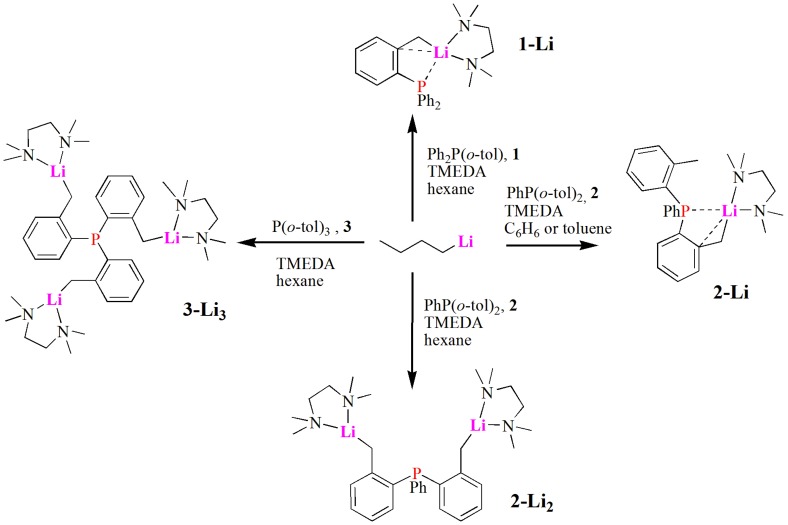
Synthesis of lithium complexes from *n*BuLi reaction of the phosphines **1**–**3** in hexane or arenic solvents.

The benzylphosphine ligands [Ph_2_P(*o*-tolyl)] (**1**), [PhP(*o*-tolyl)_2_] (**2**) and [P(*o*-tolyl)_3_] (**3**) (Scheme 1) were prepared in house using reported procedures from the suitable phosphorous chloride precursors and *o*-tolylmagnesiumbromide in the appropriate stoichiometric ratio (Scheme S1, Supplementary Materials) [10,11,12]. Lithium derivatives **1**–**3** were prepared from *n*BuLi reactions in the presence of tetramethylethylendiamine (TMEDA), as shown in Scheme 2. All complexes are yellow-orange solids extremely sensitive to environmental moisture, even more so in solution, which causes them to protonate and revert to the corresponding benzylphosphine ligand precursor. The proposed formulations are in agreement with the gathered experimental spectroscopic data: Fourier Transform Infrared (FT-IR) and ^1^H, ^7^Li, ^13^C{^1^H} and ^31^P{^1^H} nuclear magnetic resonance (NMR) spectroscopy as well as X-ray crystallographic analysis for **1-Li** and **2-Li**.

The following section describes the solid-state structures of **1-Li** and **2-Li** and is followed by a section on the Density Functional Theory (DFT) studies of a model compound. Subsequently, the solution NMR spectroscopic properties of all complexes are presented, and finally the preliminary polymerization studies are shown.

### 2.2. Solid-State Structure Determination

**Figure 1:**
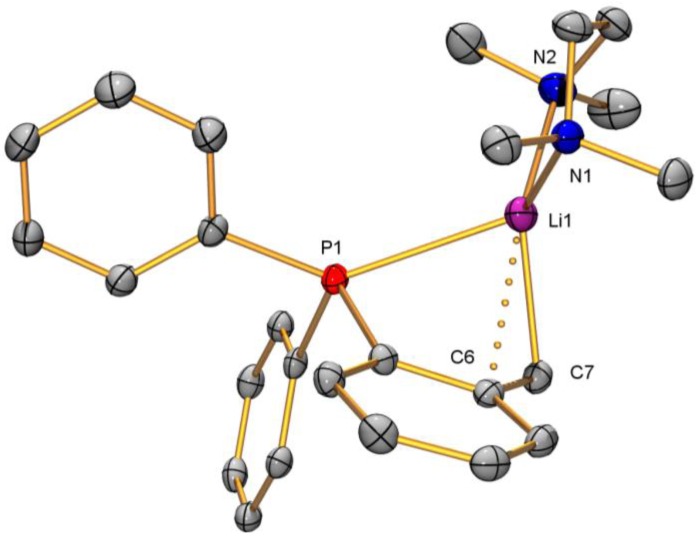
Molecular structure of **1-Li** showing thermal ellipsoids at 50% probability (all the hydrogen atoms were omitted for clarity). Selected bond lengths (Å) and angles (°): P1-Li1 2.637(3), N1-Li1 2.068(3), N2-Li1 2.069(7), C7-Li1 2.206(3), C6-Li1 2.555(3), C7-C6 1.409(2), N1-Li1-N2 88.42(12), C7-Li1-P1 76.51(10), N1-Li1-P1 116.74(13), N2-Li1-P1 118.48(13), N2-Li1-C7 136.95(16), N1-Li1-C7 122.41(15), C6-Li1-P1 64.04(7).

**Figure 2:**
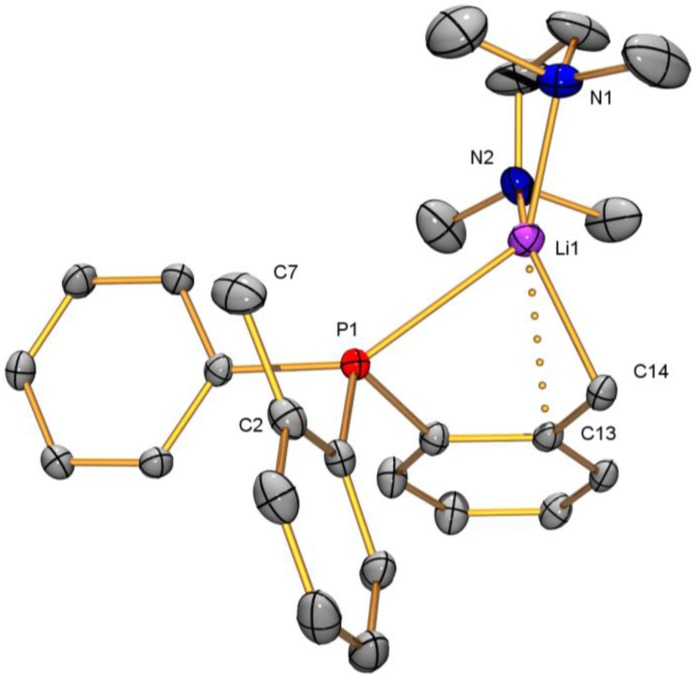
Molecular structure of **2-Li** showing thermal ellipsoids at the 50% probability level (all the hydrogen atoms were omitted for clarity). Selected bond lengths (Å) and angles (°): P1-Li1 2.680(6), N1-Li1 2.086(6), N2-Li1 2.069(7), C14-Li1 2.219(7), C13-Li1 2.536(7), C14-C13 1.409(5), C2-C7 1.523(5), N2-Li1-N1 87.9(2), C14-Li1-P1 75.75(19), N1-Li1-C14 128.3(3), N2-Li1-C14 127.8(3).

Crystals of complexes **1-Li** and **2-Li** suitable for single-crystal X-ray diffraction were isolated from slow evaporation of saturated solutions at ambient temperature. Compounds **1-Li** and **2-Li** crystallized in monoclinic space groups C2/c and P2_1_/c, respectively. The molecular structures of **1-Li** and **2-Li** are depicted in Figure 1 and Figure 2, respectively, and the crystal data are provided in the Supplementary Materials. The crystalline structures show deprotonation has taken place and a hydrogen atom has been replaced by a lithium center. The molecular structures proved these complexes are discrete mononuclear species, as is common for benzyl lithium complexes in the presence of Lewis bases [13,14]. Structures **1-Li** and **2-Li** are very similar to each other, as expected from replacing a phenyl by an *o*-tolyl group. The lithium atoms in both **1-Li** and **2-Li** are coordinated to a single TMEDA ligand in a similar fashion to other Li-TMEDA complexes reported in the literature [15,16,17].

In complex **1-Li** (see Figure 1), the two Li-N bond distances are almost equal at N1-Li1 2.068(3) Å and N2-Li1 2.069(7) Å and within the range of other reported tetracoordinated Li-TMEDA complexes. The Li bond length to the benzylic carbon C7-Li1 is 2.206(3) Å, while the corresponding bond length to the C6 *ipso* carbon of the aromatic ring is C6-Li1 2.555(3) Å, indicative of an asymmetric C-C η^2^-bonding to the Li atom, a common feature observed in benzyllithium compounds [17,18]. This interaction is also evidenced in the bent orientation of the bridged aromatic ring which arranges perpendicular to the TMEDA plane.

The Li-P bond length is 2.637(3) Å, thus significantly elongated with respect to the sum of covalent radii of P and Li (2.35–2.44 Å [19,20]) and indicative of a weak Li-P bond interaction. P to Li bonds in tertiary phosphines have been defined as lying in the range 2.50–2.68 Å in lithiumphosphinomethyl and related complexes [21]. In allyl systems bearing a basic PPh_2_ moiety, the Li-P bond was calculated to be 2.55 Å, much closer than in their NPh_2_ analogs, where the Li-N distance is 4.46 Å [22]. The angles around Li range between 76.51(10)° and 136.95(16)° if one leaves out the angles involving C7 and thus the coordination geometry can be described as a seesaw about Li. We believe the deviation from an idealized tetrahedral geometry can be credited to the establishment of the Li-P σ bond itself and the steric restrains imposed on Li-C(7) σ bond, and this deviation from ideality is less attributable to the constraining effects of the ancillary TMEDA ligand. Indeed, for the formation of the Li-P bond, the phosphine cone angle plays an important role. Even more, it should be noticed that, in the solid-state structure, C7 positions in the same plane than the aromatic ring, possibly to allow electron delocalization of the extended aromatic ring (see below for a theoretical discussion). Finally, the few other reported benzyllithium TMEDA complexes in the literature have been shown to adopt a tetrahedral geometry despite the small bite angle of the TMEDA ligand [17]. For example, in the related complex [(TMEDA)LiPhP(CHPh)_2_(CH_2_Ph)], synthesized from *n*BuLi addition to the corresponding tribenzylphosphonium chloride, a tetrahedral geometry around Li is observed with no evidence of a Li-P interaction in the solid-state structure [23]. In addition, small deviations from the idealized tetrahedral geometry are observed in the related benzyl diethylether complex [Li(Et_2_O)_2_(CHPh)PPh_2_] where no close Li-P contacts are observed [18].

The X-ray diffraction analysis of **2-Li** reveals a very similar structure to that of **1-Li** with now more dissimilar Li-N bond distances at N1-Li1 2.086(6) Å and N2-Li1 2.069(7) Å, very slightly elongated with respect to **1-Li** as expected due to the asymmetric more sterically encumbered environment resulting from the additional methyl group. The metal distances to the benzylic, C14, and the *ipso* aromatic carbon, C13, are C14-Li1 2.219(7) Å and C13-Li1 2.536(7) Å, very similar to those in **1-Li** and again resulting in a twist of the aromatic ring so that the *ipso* carbon is exposed to the metal. Thus, an asymmetric C-C η^2^ coordination of the ligand to Li can also be proposed. The Li-P distance at 2.680(6) Å is to some extent elongated with respect to **1-Li** at 2.637(3) Å but also indicative of the presence of an intramolecular bond interaction. The bond angles around Li range from 75.75(19)° to 128.3(3)° (leaving out C13) and correspond to the same type of atoms as in **1-Li**. Again, a seesaw coordination geometry about Li can be proposed. Once again, as in compound **1-Li**, the benzylic C14 positions in the same plane than the aromatic ring, possibly allowing for extended electronic delocalization. Thus, we also invoke the establishment of the Li-P intramolecular bond as well as the geometric constraints on the position of the benzylic carbon as the main factor responsible for the adoption of a seesaw coordination geometry rather than an ideal tetrahedral one.

### 2.3. DFT Studies

**Figure 3:**
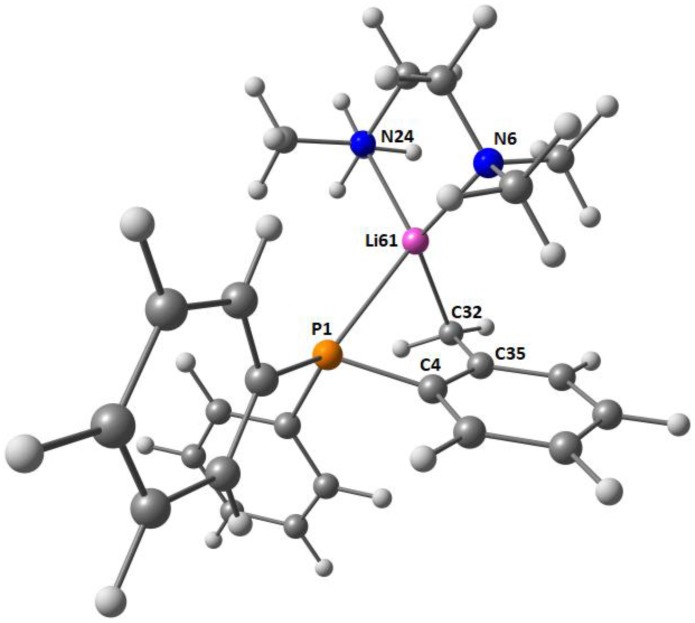
Optimized structure of **1a-Li**. Selected bond lengths (Å) and angles (°): P1-Li61 2.6383, N6-Li61 2.1093, N24-Li61 2.1112, C32-Li61 2.1275, C35-Li61 2.4864, C4-C35 1.4373, N6-Li61-N24 86.78, C32-Li61-P1 78.34, N24-Li61-P1 118.31, N6-Li61-P1 115.43, N24-Li61-C32 135.31, N6-Li61-C32 125.33, C35-Li61-P1 65.11.

To gain understanding on the bonding situation of complexes **1-Li** and **2-Li**, model **1a-Li** was computed at DFT level of theory M06/6-311+G(2d,p) [24]. The optimized geometry shown in Figure 3 is in good agreement with the X-ray structures of **1-Li** and **2-Li**; 0.08 Å is the highest difference observed between calculated and X-ray bond distances.

NBO analyses on **1a-Li** shows C32-Li61 σ-bond has a rather low 1.610 occupancy in a *p* rich hybrid orbital which is predominantly centred on the carbon atom (0.9705 C(*sp*^7.45^) + 0.2412 Li(*sp*^1.5^)). The remaining interactions observed about Li should be described as departing from an ideal Lewis structure. Second-order perturbation theory analysis of Fock matrix in NBO basis of donor–acceptor interactions show hyperconjugation of the σ orbital C32-Li61 into π* orbital of C35-C4 (61.43 kcal mol^−1^). This π* orbital is substantially located on C35 (62.6%), which is related to the *ipso* C interaction observed in the X-ray structures of **1-Li** and **2-Li** (C6 in **1-Li** and C13 in **2-Li**). These findings are in agreement with the observation in the experimental structures of the benzylic carbon lying in the same plane than the aromatic ring to which it is bonded. Since the benzylic carbon is disposed to allow extended electronic delocalization of the aromatic ring, its position is subsequently constrained and thus its bonding angles to lithium. Thus, the adoption of seesaw geometries around Li are justified. The P lone pair (*sp*^1.26^) is partially delocalized on an available *sp*^2^ orbital on Li (*sp*^2.04^); this is the most important interaction in the donor–acceptor analysis (74.33 kcal mol^−1^). An additional bonding contribution of the phosphorous atom to Li stems from additional delocalization of σ P1-C4 orbital into the aforementioned *sp*^2^ orbital on Li. N atoms of TMEDA bind Li atom with two interactions; σ N-C orbital delocalization in partially occupied idealized *sp*^3^ orbital on Li and delocalization of the lone pair of N into the same *sp*^3^ orbital. The contribution of both interactions is ≈20 kcal mol^−1^ (σ_N-C_ into sp_Li_^3.94^ 9.36 and 9.75 kcal mol^−1^; LP_N_sp^6^ into sp_Li_^3.94^ 11.65 and 10.8 kcal mol^−1^).

### 2.4. Solution NMR Spectroscopy

The main features observed in the NMR spectra of the complexes are described below, whereas the complete set of spectra are shown in the Supplementary Materials. ^1^H, ^7^Li, ^13^C{^1^H} and ^31^P{^1^H}, NMR spectra of all four lithium complexes measured in C_6_D_6_ at 298 K demonstrate the purity of each compound. In all cases, the ^1^H and ^13^C{^1^H} NMR spectra exhibit only one set of signals for the phenyl, benzylic, methylene and methyl groups of TMEDA with integrations consistent with the proposed formulations. For example, the ^1^H NMR spectra of **1-Li** shows a singlet signal at δ 2.35 corresponding to the benzylic CH_2_Li group while at δ 1.91 and 1.85 two singlets for the methyl and methylene hydrogens of TMEDA are observed. Correspondingly, in the ^13^C{^1^H} NMR spectrum a doublet at δ 43.71 (*^3^J_P_*_,*C*_ = 17.5 Hz) and a singlet at δ 45.57 are assigned respectively to the benzylic [25] and TMEDA methyl carbons whilst the signal at δ 56.87 is attributed to the TMEDA methylene carbon.

**Scheme 3:**
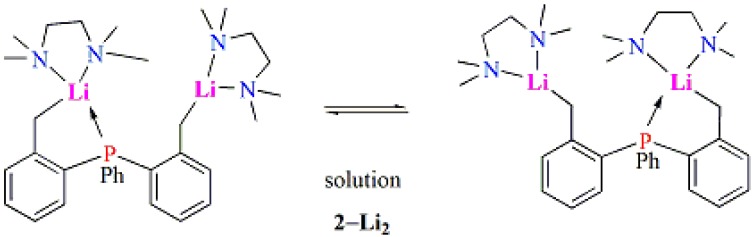
Equilibrium involving the coordination and de-coordination of the phosphorous atom to each of the lithium atoms in complex **2-Li_2_**, which accounts for a single set of broad resonances at room temperature for the benzylic hydrogens and carbons as well as the rest of the spectral signals otherwise chemically inequivalent.

In the ^1^H and ^13^C{^1^H} NMR spectra of complex **2-Li_2_**, a broad signal for the two benzylic groups is observed. This may be due to a fluxional behavior involving the coordination and de-coordination of the phosphorous atom to the lithium centers, as shown in Scheme 3.

In each of the ^7^Li NMR spectra of the four complexes, only one broad signal is observed at δ 1.59 for **1-Li**, 1.94 for **2-Li**, 0.42 for **2-Li_2_**, and 0.89 for **3-Li_3_**, due to unresolved coupling to the ^31^P nuclei at ambient temperature and evidencing magnetically equivalent nuclei in the cases of **2-Li_2_** and **3-Li_3_**.

**Figure 4:**
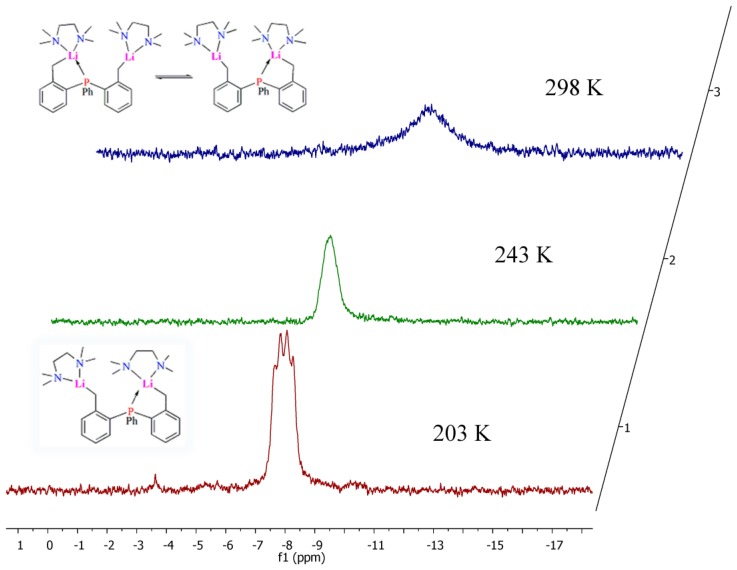
^31^P{^1^H} NMR spectra in toluene-d_8_ of **2-Li_2_** at three different temperatures.

**Figure 5:**
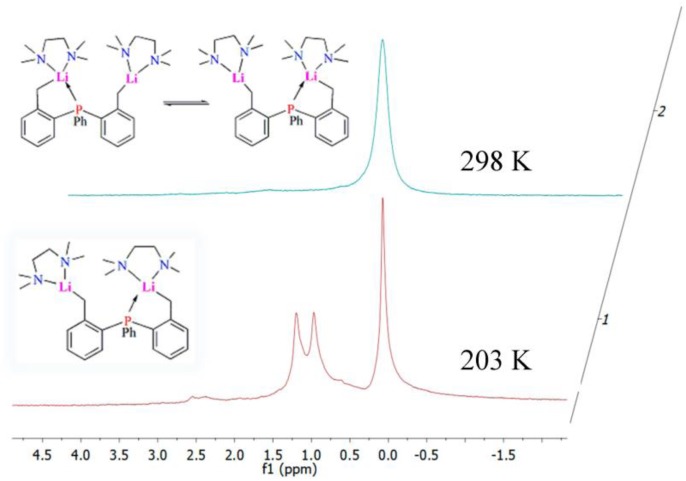
^7^Li NMR spectra in toluene-d_8_ of **2-Li_2_** at two different temperatures showing at the slow exchange regime two signals for the two lithium nuclei as a doublet at δ 1.08 and singlet at δ 0.08.

In addition, for the four complexes, singlet signals are observed in their ^31^P{^1^H} NMR spectra at ambient temperature at δ: −15.30 (w_1/2_ = 4.0 Hz) for **1-Li**, −21.13 (w_1/2_ = 6.1 Hz) for **2-Li**, −12.83 (w_1/2_ = 42 Hz) for **2-Li_2_** and −33.08 (w_1/2_ = 56 Hz) for **3-Li_3_**. Thus, whereas the ^31^P{^1^H} NMR spectral signals of **2-Li_2_** and **3-Li_3_** were broad, the compounds bearing only one lithium atom **1-Li** and **2-Li** exhibited sharp signals. Since at room temperature it was not possible to determine the value of the coupling between phosphorus and lithium J*_PLi_* in the ^31^P{^1^H} NMR spectrum, we pursued a VT-NMR experiment in toluene-d_8_ of an NMR tube containing **2-Li_2_** (Figure 4). The ^31^P{^1^H} NMR spectrum showed at 203 K the signal belonging to **2-Li_2_** resolving into a pseudo-quartet of approximate intensities 1:1:1:1 from which a ^1^*J*_PLi_ of 42 Hz was determined. At the slow exchange regime, the 1:1:1:1 ^31^P{^1^H} NMR signal of **2-Li_2_** is in agreement with a bonding interaction of the ^31^P nucleus to a single ^7^Li (S = 3/2) at the time. In a similar manner, at 203 K the ^7^Li NMR spectrum of **2-Li_2_** shows the ambient temperature broad signal at δ 0.42 resolving into a sharper signal at δ 0.08 and a doublet at δ 1.08 with a coupling constant ^1^*J*_PLi_ = 42 Hz (Figure 5), the same value determined in the ^31^P{^1^H} NMR spectrum. These data are in agreement with two types of lithium nuclei at the slow exchange regime, only one of them coupled to phosphorous. Similar experiments on **1**-**Li** did not allow for the measurement of any ^1^*J*_PLi_ coupling in solution, in agreement with a weaker intramolecular P-Li interaction in **1-Li** in solution with respect to **2-Li_2_**.

**Scheme 4:**
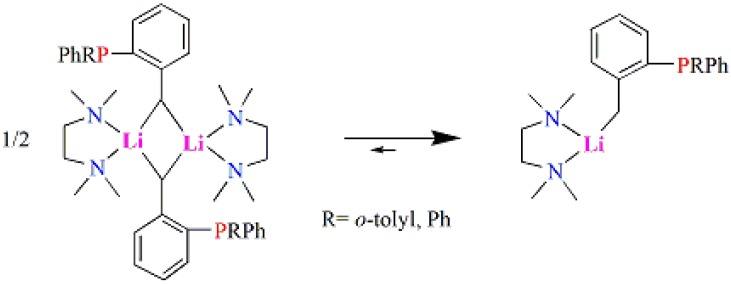
Proposed structures for **1-Li** and **2-Li** in solution. The monomeric structures would be most favored due to strong steric hindrance caused by the bulky substituents on the P atoms.

Although it is widely known that many organolithium species exist in solution as dimers or oligomers generated upon the formation of C-Li bridges [17,26], the fact that in the solid state the molecular structures of crystals of **1-Li** and **2-Li** are monomeric, even when generated from non-coordinating hexane solutions, is in agreement with the preservation of the monomeric structures in solution. Thus, the equilibrium shown in Scheme 4 would be so largely shifted to the right to the point that we observed no evidence of the dimeric structures by NMR spectroscopic means.

At this point, attention should be drawn to the fact that the nature of the solvent media is determinant in the formation of complex **2-Li_2_**. Indeed, when the reaction of precursor **2** with *n*BuLi is carried out in either benzene or toluene, formation of **2-Li** is preferred and a very large excess of *n*BuLi (>12 equiv) is required to effect total conversion to **2-Li_2_** (see Supplementary Materials). In contrast, performing the reaction with 2 or more equiv of *n*BuLi in hexane leads to total conversion to **2-Li_2_**. This is explained in terms of the competition of the arenic solvent for deprotonation by *n*BuLi and it is evidenced in the formation of PhLi which was unambiguously detected (NMR and X-ray diffraction) as a by-product when the reaction was carried out in benzene.

### 2.5. Preliminary Polymerization Studies

#### 2.5.1. Solvent-Free Ring Opening Polymerizaton of *ε*-Caprolactone

Complexes **1-Li**, **2-Li**, **2-Li_2_** and **3-Li_3_** were preliminarily tested in the solvent-free ROP of *ε*-CL at room temperature. The results of these initial polymerization studies are summarized in Table 1.

As it can be appreciated, all four complexes showed to be highly active in the polymerization of *ε*-CL exhibiting very high conversions (94–99%) after short times of 1–6 min. As a result, in Table 1, conversion values and polymer parameters are not given at the same reaction times for the four different catalysts. Conversion times were chosen in Table 1 as the times in which the reaction had apparently finished by complete lack of liquid phase. The catalytic activity is fairly similar for all four complexes. However, the determined molecular weights *M_n_*_,*obsd*_ of the synthesized polymers are very dissimilar and in general fairly low (7400 < *M_n_*_,*obsd*_ < 32,700). Moreover, the calculated (*M_n_*_,*calcd*._) and experimental (*M_n_*_,*obsd*_) values for the number-average molecular weight were very dissimilar, suggesting lack of control of the polymerization reaction. As a result, the dispersity values were also widespread (between 1.70 and 2.97), suggesting that polymerization side reactions such as intra- and intermolecular transesterifications may take place.

#### 2.5.2. Solvent-Free Ring-Opening Polymerization of *rac*-LA

Complexes **1-Li**, **2-Li**, **2-Li_2_** and **3-Li_3_** were also preliminarily tested in the solvent-free polymerization of *rac*-LA at 140 °C. The results of the polymerization are given in Table 2.

The isolated polymers had molecular weights between 3400 < *M_n_*_,*obsd*_ < 12,100. The calculated (*M_n_*_,*calcd*._) and experimental (*M_n_*_,*obsd*_) values for the number-average molecular weight of the synthesized polymers were fairly similar in the case of **1-Li** (entry 1, Table 2), suggesting some control in the polymerization reaction for that initiator, although polymer molecular weight distribution is broad (*M**_w_**/M_n_* = 1.83) [5,27,28]. Under the conditions investigated, the catalysts show low stereocontrol giving predominantly atactic polymers with some heterotactic enrichment for **1-Li** and **2-Li_2_** (*P_r_* ~ 0.6).

## 3. Materials and Methods

### 3.1. General Considerations

All experiments were performed under an argon atmosphere using standard Schlenk techniques or inside an UNIlab glove box workstation (MBraun, Stratham, NH, USA) using copper oxide as catalyst (PuriStar Catalyst R3-11G) and molecular sieves Molsiv Adsorbents 13X 1/8). Toluene, benzene, hexane and pentane were either dried and distilled from sodium using benzophenone ketyl as an indicator or purified over an solvent purification column system (MB-SPS-800, MBraun, Stratham, NH, USA). In either case, they were degassed prior to use. Benzene-d_6_ and toluene-d_8_ were degassed via three freeze–pump–thaw cycles and stored over either molecular sieves or a freshly prepared potassium mirror in an ampoule fitted with a J. Young’s valve. 2-bromotoluene, Ph_2_PCl, PhPCl_2_, PCl_3_ and *n*BuLi were purchased from Sigma Aldrich Mexico (Sigma Aldrich Química, Toluca, Estado de México, Mexico) and used as received. Phenyllithium, Ph_2_P(*o*-tolyl), PhP(*o*-tolyl)_2_ and P(*o*-tolyl)_3_ were prepared by a modified literature method. ^1^H, ^7^Li, ^31^P{^1^H} and ^13^C{^1^H} NMR spectra in solution were recorded on Bruker Avance 500 MHz (Bruker Daltonics Inc., Billerica, MA, USA), Varian Inova 400 MHz, Varian VNMRS 700 MHz or Varian Mercury 200 MH spectrometers (Varian Inc., Palo Alto, CA, USA; now Agilent Technologies NMR). ^1^H and ^13^C NMR chemical shifts were determined by reference to the residual solvent peaks. ^31^P and ^7^Li NMR spectra were referenced externally using H_3_PO_4_ and LiCl in D_2_O, respectively. IR data were recorded as KBr pellets on a Nicolet 6700 FT-IR spectrometer and are reported in cm^−1^. Crystals of **1-Li** and **2-Li** were fixed in a glass fiber and measured at 100 K. X-ray intensity data were collected using the program CrysAlisPro [29] on a four-circle SuperNova, Dual EosS2 CCD diffractometer (Agilent Technologies XRD Products, Yarnton, Oxfordshire, UK) with monochromatic Cu-Kα radiation (λ = 1.54184 Å). Cell refinement, data reduction, incident beam, decay and absorption corrections were carried out with the use of the program CrysAlisPro [29]. Using Olex 2 [30], the structure was solved by direct methods with the program SHELXT and refined by full-matrix least-squares techniques with SHELXL [31,32,33]. Further details of experimental and structure analyses are given in CCDC 1586330 and 1586331, respectively, and in the Supplementary Materials. All hydrogen atoms were generated in calculated positions and constrained with the use of a riding model. The final model involved anisotropic displacement parameters for all non-hydrogen atoms. DFT computations were performed on model **1a-Li**. Geometry optimization of **1a-Li** was carried out in the gas phase with the M06 level of theory as implemented in the Gaussian09 series of programs [34]. The all electron basis set 6-311+G(2d,p) was applied to all atoms in **1a-Li**. Frequency calculations in the harmonic approximation performed at the same level of theory were used to verify that the optimized structure of **1a-Li** is located in a global minimum of the potential energy hypersurface.

### 3.2. Synthesis of Compounds

#### 3.2.1. Synthesis of Complex [Ph_2_P(C_6_H_4_CH_2_Li·TMEDA)] (**1-Li**)

To a Schlenk flask charged with a solution of [Ph_2_P(*o*-tolyl)] (1 g, 3.61 mmol) in hexane (40 mL), tetramethylethylenediamine, TMEDA (0.54 mL, 3.61 mmol) and *n*BuLi (1.44 mL of a hexane solution, 3.61 mmol) were added at −78 °C with stirring. The reaction mixture was then kept at 0 °C for 16 h, after which it was filtered and dried under reduced pressure yielding an extremely air and moisture sensitive yellow-orange solid. Crystals for the X-ray experiment were obtained from benzene. Yield: (1.24 g, 86%). m.p.: 146–148 °C. ^1^H NMR (500 MHz, C_6_D_6_, 298 K): δ 7.57 (m, 4H, C*H arom*), 7.36 (m, 1H, C*H arom*), 7.16 (m, 1H, C*H arom*), 7.09 (m, 3H, C*H arom*), 7.03 (m, 2H, C*H arom*), 6.99 (m, 1H, C*H arom*), 6.73 (m, 1H, C*H arom*), 6.19 (m, 1H, C*H arom*), 2.35 (br, 2H, C*H*_2_Li), 1.85 (s, 12H, C*H*_3_N), 1.65 (s, 4H, C*H*_2_N). ^13^C{^1^H} NMR (100.68 MHz, C_6_D_6_, 298 K): δ 43.71 (d, *C*H_2_-Li, *^3^J_P_*_,*C*_ = 17.5 Hz), 45.57 (s, *C*H_3_N), 56.87 (s, *C*H_2_N), 108.12 (s, *C*H, *o-tolyl*), 120.68 (s, *C*H, *o-tolyl*), 127.94 (s, *C*H, *Phenyl*), 128.03 (d, *C*H, *^3^J_P_*_,*C*_ = 7.4 Hz, *Phenyl*), 128.3 (s, *C*H, *o-tolyl*), 128.80 (s, *C*H, *Phenyl*), 132.79, (s, *C*H, *o-tolyl*), 133.9 (d, *C*H, *^2^J_P_*_,*C*_ = 20.1 Hz, *o-tolyl*), 134.2 (s, *C*H, *^2^J_P_*_,*C*_ = 17.5 Hz, *Phenyl*), 136.27 (d, *C-P*, *^1^J_P_*_,*C*_ = 12.57 Hz, *o-tolyl*), 136.66 (d, *C-P*, *^1^J_P_*_,*C*_ = 11.3 Hz, *Phenyl*), 157.5 (d, *C-P*, *^1^J_P_*_,*C*_ = 26.39 Hz, *o-tolyl*). ^31^P{1H} NMR (161.92 MHz, C_6_D_6_, 298 K): δ −15.30 (s, w_1/2_ 4 Hz).). ^7^Li NMR (155.45 MHz, C_6_D_6_, 298 K): δ 1.59 (s). IR (KBr/cm^−1^): 3059 (m), 2962 (m), 1585 (m), 1470 (m), 1434 (m), 1258 (m), 1176 (m), 1088 (m), 1026 (m), 803 (m), 742 (s), 693 (s), 551 (m), 511 (m).

#### 3.2.2. Complex [Ph(*o*-tolyl)P(C_6_H_4_CH_2_Li·TMEDA)] (**2-Li**)

To a Schlenk flask charged with a solution of [PhP(*o*-tolyl)_2_] (0.5 g, 1.74 mmol) in benzene (20 mL), TMEDA (0.52 mL, 3.48 mmol) and *n*BuLi (1.4 mL of a hexane solution, 3.48 mmol) were added at −78 °C with stirring (these conditions were used in a failed attempt at preparing **2-Li_2_** but employing a 1:1:1 molar ratio of [PhP(*o*-tolyl)_2_], TMEDA and *n*BuLi also leads to **2-Li**). The reaction mixture was then kept at 0 °C for 16 h, after which it was filtered off and dried under reduced pressure. An extremely air sensitive orange solid was isolated. Crystals for the X-ray experiment were obtained from a concentrated benzene solution. Yield: (0.63 g, 89%). ^1^H NMR (700 MHz, C_6_D_6_, 298 K): δ 7.32 (m, 2H, C*H arom*), 7.17 (s, 2H, C*H arom*), 7.01 (s, 4H, C*H arom*), 6.98 (m, 3H, C*H arom*), 6.87 (m, 2H, C*H arom*), 2.37 (d, 3H, *J_P_*_,*H*_ = 0.87 Hz, C*H*_3_ *o-tolyl*), 2.31 (s, 4H, C*H*_2_N), 2.08 (s, 12H, C*H*_3_N). ^13^C{^1^H} NMR (176.01 MHz, C_6_D_6_, 298 K): δ 23.54 (d, *CH*_3_ *o-tolyl*, *^3^J_P_*_,*C*_ = 22.75 Hz), 48.27 (s, *C*H*_3_*N), 60.65 (s, *C*H*_2_*N), 128.80 (s, *C*H, *o-tolyl*), 130.58 (s, *C*H *Phenyl*), 130.82 (s, *C*H *Phenyl*), 131.19 (s, *C-H Phenyl*), 131.24 (s, *C*H *Phenyl*), 131.29 (s, *C*H, *o-tolyl*), 132.75 (s, *C*H *o-tolyl*, *^3^J_P_*_,*C*_ = 5.28 Hz), 135.61 (s, *C*H, *o-tolyl*), 137.00 (d, *C*H *Phenyl*, *^2^J* = 19.3 Hz), 138.20 (s, *C-P o-tolyl*, *^1^J_P_*_,*C*_ = 12.3 Hz), 138.56 (d, *C-P Phenyl*, *^1^J_P_*_,*C*_ = 10.56 Hz), 145.50 (d, *C-P*, *o-tolyl, ^1^J_P_*_,*C*_ = 24.60 Hz). ^31^P{^1^H} NMR (161.92 MHz, C_6_D_6_, 298 K): δ −21.13 (s, w_1/2_ 6 Hz). ^7^Li NMR (194.3 MHz, C_6_D_6_, 298 K): δ 1.94 (s). IR (KBr/cm^−1^): 3044.5 (m), 2964.0 (m), 2845.0 (m), 1591.2 (m), 1571.0 (m), 1471.3 (m), 1445.7 (m), 1429.4 (m), 1265.4 (m), 1088.4 (s), 1027.3 (s), 802.1 (m), 741.0 (s), 702.4 (s), 547.9 (s).

#### 3.2.3. Complex [PhP(C6H4CH2Li·TMEDA)2] (**2-Li2**)

To a Schlenk flask charged with a solution of [PhP(*o*-tolyl)_2_] (1 g, 3.44 mmol) in hexane (40 mL), TMEDA (1.0 mL, 6.88 mmol) and *n*BuLi (2.8 mL of a hexane solution, 6.88 mmol) were added at −78 °C with stirring. The reaction mixture was then kept at 0 °C for 16 h. It yielded an extremely air sensitive orange solid, which was filtered off and dried under reduced pressure. Yield: (1.47 g, 80%). m.p.: decomposes above 135 °C. ^1^H NMR (500 MHz, C_6_D_6_, 298 K): δ 8.11 (m, 2H, C*H arom*), 7.31 (m, 2H, C*H arom*), 7.26 (m, 2H, C*H arom*), 7.12 (m, 1H, C*H arom*), 6.91 (m, 2H, C*H arom*), 6.63 (m, 2H, C*H arom*), 6.07 (m, 2H, C*H arom*), 2.51 (s, 4H, C*H*_2_Li), 1.92 (s, 24H, C*H*_3_N), 1.72 (s, 8H, C*H*_2_N). ^13^C{^1^H} NMR (125.72 MHz, C_6_D_6_, 298 K): δ 45.32 (s, overlapped *C*H_3_N and *C*H*_2_*Li), 56.30 (C*H*_2_N), 105.18 (br, *CH*, *o-tolyl*), 119.16 (b, *CH*, *o-tolyl*), 126.86 (s, *CH Phenyl*), 127.79 (d, *CH Phenyl*, *^3^J_P_*_,*C*_ = 6.8 Hz), 128.19 (b, *CH*, *o-tolyl*), 134.08 (d, *CH Phenyl ^2^J_P_*_,*C*_ = 15.3 Hz), 134.71 (d, *C-P Phenyl*, *^1^J_P_*_,*C*_ = 18.1 Hz), 134.06 (b, *CH*, *o-tolyl*), 155.77 (b, *CH*, *o-tolyl*). ^31^P{^1^H} NMR (202.4 MHz, C_6_D_6_, 298 K): δ −12.83 (s br, w_1/2_ = 42 Hz). ^31^P{^1^H} NMR (161.9 MHz, toluene-d_8_, 203 K): δ −7.96 (~1:1:1:1 quartet, ^1^*J*_PLi_ = 42 Hz). ^7^Li NMR (194.3 MHz, toluene-d_8_, 293 K): δ 0.80 (br, w_1/2_ = 31 Hz). ^7^Li NMR (194.3 MHz, toluene-d_8_, 203 K): δ 0.08 (s), 1.08 (d, ^1^*J*_PLi_ = 42 Hz). IR (KBr/cm^−1^): 3049.3 (m), 2982.9 (m), 2957.0 (m), 2869.7 (m), 2829.7 (m), 2788.9 (m), 1574.7 (s), 1460.6 (s), 1416.1 (m), 1299.1 (m), 1273.7 (m), 1096.8 (m), 1021.45 (s), 946.3 (m), 792.2 (m), 749.2 (m).

#### 3.2.4. Complex [P(C6H4CH2Li·TMEDA)3] (**3-Li3**)

To a Schlenk flask charged with a solution of [P(*o*-tolyl)_3_] (0.64 g, 2.13 mmol) in hexane (40 mL), TMEDA (0.95 mL, 6.40 mmol) and *n*BuLi (2.5 mL of a 2.5 M solution in hexane, 6.40 mmol) were added at −78 °C with stirring. The reaction mixture was then kept at 0 °C for 16 h. It yielded an extremely air sensitive orange solid, which was filtered off and dried under reduced pressure. Yield: (1.19 g, 85%). ^1^H NMR (500 MHz, C_6_D_6_, 298 K): δ 7.04 (m, 3H, C*H arom*), 6.87 (m, 3H, C*H arom*), 6.63 (m, 3H, C*H arom*), 5.93 (m, 3H, C*H arom*), 2.69 (br, 6H, C*H*_2_Li), 2.02 (s, 36 H, C*H*_3_N), 1.88 (s, 12 H, CH_2_N). ^31^P{^1^H} NMR (202.4 MHz, C_6_D_6_, 298 K): δ −33.08 (s br, w_1/2_ 56 Hz). ^7^Li NMR (194.3 MHz, C_6_D_6_, 298 K): δ 0.89 (s).

### 3.3. Polymerization Procedure

#### 3.3.1. Solvent-Free Ring-Opening Polymerization of ε-CL

The polymerization reactions of *ε*-CL employing each of the lithium complexes **1-Li**, **2-Li**, **2-Li_2_** and **3-Li_3_** were performed in Schlenk tubes with magnetic stirring under an argon atmosphere at room temperature. The molar ratio of monomer to catalyst employed was 100:1. Liquid *ε*-CL (1 mL) was placed in the Schlenk tube under argon, degassed and subsequently, the corresponding molar amount of the lithium complex was added via a glass tube from a second Schlenk flask. The polymerization reactions took place within minutes of mixing leading to the reaction mixture being converted to off-white solids. The polymerization was quenched with a few drops of water. The resulting polymers were dissolved in dichloromethane, an aliquot of the solution was dried, dissolved in CDCl_3_ and analyzed by ^1^H NMR spectroscopy to determine the percentage of conversion. Isolation of the polymers was effected by addition of cold methanol and filtration. The solids were dried in vacuo for at least 12 h. The polymers were afterwards characterized by gel permeation chromatography (GPC) and NMR spectroscopy.

#### 3.3.2. Solvent-Free Ring-Opening Polymerization of rac-LA

The lithium complexes **1-Li**, **2-Li**, **2-Li_2_** and **3-Li_3_** were also tested as initiators for the polymerization of *rac*-LA carried out without solvent in Ace pressure reactors with stirring under argon atmosphere at 140 °C. Weighted solids *rac*-LA and lithium catalyst were loaded in the MBraun glovebox and stirred at room temperature for 5 min in an Ace pressure reactor at room temperature. After this time, the vessel was immersed in a hot oil bath with the temperature set to 140 °C to ensure all *rac*-LA was in liquid state as soon as the immersion took place. The polymerization was quenched with a few drops of water. The resulting polymers were dissolved in dichloromethane, an aliquot of the solution was dried, dissolved in CDCl_3_ and analyzed by ^1^H NMR spectroscopy to determine the percentage of conversion. Isolation of the polymers was effected by addition of cold methanol and filtration. The solids were dried in vacuo for at least 12 h. The polymers were afterwards characterized by gel permeation chromatography (GPC) and NMR spectroscopy.

## 4. Conclusions

Four lithium complexes derived from benzylphosphines were isolated and characterized spectroscopically. In the solid state, **1-Li** and **2-Li** were found to be monomeric comprising seesaw lithium atoms. An asymmetric C-C η^2^-coordination of the benzyl fragment to lithium was observed as well as an intramolecular Li-P bond interaction, which was further characterized by DFT means. It was found by second-order perturbation theory analysis there exists hyperconjugation of the Li-C benzylic σ orbital into a π* orbital of the extended aromatic system substantially related to the Li-*ipso* C interaction observed. The main contribution to the intramolecular Li-P bond was found to be the P lone pair partially delocalized on an available *sp*^2^ orbital on Li. A preliminary study shows compounds **1-Li**, **2-Li**, **2-Li_2_** and **3-Li_3_** are active catalysts in the solvent free ring-opening polymerization of *ε*-caprolactone and *rac*-lactide. High conversions to polycaprolactones were obtained in short periods of time: 1–6 min at 25 °C when using a molar ratio monomer/catalyst of 100. On the other hand, all four complexes behave as moderately good initiators for the ROP of *rac*-LA showing high conversions to polylactides at 140 °C.

## Figures and Tables

**Table 1:** Ring opening polymerization (ROP) of *ε*-caprolactone (*ε*-CL) catalyzed by lithium complexes. Cat = catalyst, *t* = time.

Entry	Cat	*t* (Minute)	Conversion ^a^ (%)	*M_n_*_,*calcd*._*^,^* ^b^ × 10^3^	*M_n_*_,*obsd*_^c^ × 10^3^	*M_w_/M_n_*
1	1-Li	2	99	11.3	32.7	1.70
2	2-Li	2	94	10.7	7.4	2.69
3	2-Li_2_	6	98	11.2	28.4	2.97
4	3-Li_3_	1	98	11.2	16.0	2.89

The reactions were run in 1 mL of *ε*-CL using an initial molar ratio [*ε*-CL]_0_/[Cat]_0_ = 100 at ambient temperature for the times indicated above. ^a^ Determined by ^1^H NMR spectroscopy. ^b^ Calculated according to *M_n_*_,*calcd*._ = ([*ε*-CL]_o_/[Cat.]_o_) × 114.14 × conversion %. ^c^ The *M_n_*_,*obsd*_ value was calculated considering Mark-Houwink’s correction of *M_n_*_,*obsd*_ = 0.56*M_n_*^GPC^, where *M_n_*^GPC^ was determined by GPC in THF relative to polystyrene standards (see Table S14 of the Supplementary Materials for yields of the isolated polymers).

**Table 2:** ROP of *rac*-lactide (*rac*-LA) catalyzed by lithium complexes. Cat = catalyst, *P_r_* = heterotactic probability.

Entry	Cat	Conversion ^a^ (%)	*M_n_*_,*calcd*._^b^ × 10^3^	*M_n_*_,*obsd*_^c^ × 10^3^	*M_w_/M_n_*	*P_r_* ^d^
1	1-Li	93	13.4	12.1	1.83	0.61
2	2-Li	95	13.7	3.4	2.53	0.52
3	2-Li_2_	97	14.0	5.3	2.61	0.58
4	3-Li_3_	98	14.1	6.7	2.24	0.53

The reactions were run using an initial molar ratio [*rac*-LA]_0_/[Cat]_0_ = 100 at 140 °C for 1 h. ^a^ Determined by ^1^H NMR spectroscopy. ^b^ Calculated according to *M_n_*_,*calcd*._ = ([*rac*-LA]_0_/[Cat]_0_) × 144.13 × conversion %. ^c^ The *M_n_*_,*obsd*_ value was calculated considering Mark-Houwink’s correction of *M_n_*_,*obsd*_ = 0.58*M_n_*^GPC^, where *M_n_*^GPC^ was determined by GPC in THF relative to polystyrene standards. ^d^ Determined by analysis of *rmr* tetrad signal in the methine region of the homonuclear-decoupled ^1^H NMR spectra (see Supplementary Materials for further details of the calculations).

## Supplementary Materials

The following are available online. Full NMR and IR spectroscopic characterization of all complexes, X-ray diffraction data, Cartesian coordinates for DFT studies of **1a-Li**, and additional scheme for the synthesis of the phosphine precursors. NMR spectra of polymers and description of the procedure for the calculation of *M_n*,*calcd*.*_* values. The X-ray diffraction structures have been deposited at the Cambridge Crystallographic Data Centre (CCDC): **1-Li** (1586330) and **2-Li** (1586331).
