# Supplementary material for: Lithium Complexes Derived of Benzylphosphines: Synthesis, Characterization and Evaluation in the ROP of rac-Lactide and ε-Caprolactone
Source: Molecules. 2017 Dec 30;23(1):82. doi: 10.3390/molecules23010082 (PMC6017396; doi:10.3390/molecules23010082)
Supplement: Supplementary file 1 [file molecules-23-00082-s001.pdf]

Electronic supplementary information

for

Lithium complexes derived of benzylphosphines: Synthesis, characterization and evaluation in the ROP of *rac*-lactide and ε-caprolactone

Ernesto Rufino-Felipe, Miguel A. Muñoz-Hernández and Virginia Montiel-Palma*

Centro de Investigaciones Químicas, Instituto de Investigación en Ciencias Básicas y Aplicadas, Universidad Autónoma del Estado de Morelos, Av. Universidad 1001, Col. Chamilpa, Cuernavaca, Morelos 62209 México. Tel.:+ 52 777 3297997

E-mail: vmontielp@uaem.mx

Contents

1. NMR spectroscopic data for all lithium complexes 4

Figure S1. ^{1}H NMR spectrum (400 MHz, C_6D_6, 298 K) of complex **1-Li**. 4

Figure S2. ^{31}P{^{1}H} NMR spectrum (161.92 MHz, C_6D_6, 298 K) of complex **1-Li**. 4

Figure S3. DEPTQ NMR spectrum (100.68 MHz, C_6D_6, 298 K) of complex **1-Li**. 5

Figure S4. ^{7}Li NMR spectrum (155.45 MHz, C_6D_6, 298 K) of complex **1-Li**. 6

Figure S5. ^{31}P{^{1}H} NMR spectrum (202.40 MHz, C_7D_8) at variable temperatures of complex **1-Li**. 7

Figure S6. ^{1}H NMR spectrum (700 MHz, C_6D_6, 298 K) of complex **2-Li**. 8

Figure S7. ^{31}P{^{1}H} NMR spectrum (161.92 MHz, C_6D_6, 298 K) of complex **2-Li**. 9

Figure S8. ^{7}Li NMR spectrum (155.45 MHz, C_6D_6, 298 K) of complex **2-Li**. 9

Figure S9. ^{13}C{^{1}H} NMR spectrum (700 MHz, C_6D_6, 298 K) of complex **2-Li**. 10

Figure S10. HSQC ^{1}H-^{13}C NMR spectrum (700 MHz, C_6D_6, 298 K) of complex **2-Li.** 11

Figure S11. ^{1}H NMR spectrum (400 MHz, C_6D_6, 298 K) of complex **2-Li$_2$**. 11

Figure S12. ^{31}P{^{1}H} NMR spectrum (161.92 MHz, C_6D_6, 298 K) of complex **2-Li$_2$**. 12

Figure S13. ^{31}P{^{1}H} NMR spectra for the titration of [PhP(o-tolyl)2] with excess *n*BuLi, evidencing the formation of complexes **2Li** and **2-Li$_2$** (161.92 MHz, C_6D_6, 25 ℃). 13

Figure S14. ^{7}Li NMR spectrum (155.45 MHz, C_6D_6, 298 K) of complex **2-Li$_2$** 14

Figure S15. ^{13}C{^{1}H} NMR spectrum (125.72 MHz, C_6D_6, 25 ℃) of complex **2-Li$_2$**. 15

Figure S16. ^{31}P{^{1}H} NMR spectrum (202.40, toluene-d_8) of complex **2-Li$_2$** at given temperatures. 16

Figure S17. ^{7}Li NMR spectrum at 298 K and 203 K (194.32 MHz, C_7D_8) of complex **2-Li$_2$** 17

Figure S18. ^{1}H NMR spectrum (400 MHz, C_6D_6, 25 ℃) of complex **3-Li$_3$** 17

Figure S19. ^{31}P{^{1}H} NMR spectrum (161.92 MHz, C_6D_6, 298 K) of complex **3-Li$_3$**. 18

Figure S20. ^{7}Li NMR spectrum (194.32 MHz, C_6D_6, 298 K) of complex **3-Li$_3$**. 18

2. FT IR spectra 19

Figure S21. Infrared spectrum of **1-Li** (KBr disc) 19

Figure S22. Infrared spectrum of **2-Li** (KBr disc) 20

Figure S23. Infrared spectrum of **2-Li$_2$** (KBr disc). 21

Figure S24. Infrared spectrum of **2-Li$_3$** (KBr disc). 22

3. X-ray diffraction data 23

3.1 X-ray diffraction data of complex **1-Li** 23

Table S 1. Crystal data and structure refinement for **1-Li**. 23

Table S 2. Fractional Atomic Coordinates (×104) and Equivalent Isotropic Displacement Parameters (Å2×103) for **1-Li**. 23

Table S 3. Anisotropic Displacement Parameters (Å2×103) for **1-Li**. 24

Table S 4. Bond Lengths for **1-Li**. 25

Table S 5. Bond Angles for **1-Li**. 25

Table S 6. Torsion Angles for MM89a. 26

Table S 7. Hydrogen Atom Coordinates (Å×104) and Isotropic Displacement Parameters (Å2×103) for **1-Li**. 27

3.2 X-ray diffraction data of complex **2-Li** 29

Table S 8. Crystal data and structure refinement for 2-Li........ 29

Table S 9. Fractional Atomic Coordinates (×104) and Equivalent Isotropic Displacement Parameters (Å2×103) for MM122........ 29

Table S 10. Anisotropic Displacement Parameters (Å2×103) for **2-Li**........ 30

Table S 11. Bond Lengths for **2-Li**........ 31

Table S 12. Bond Angles for **2-Li**........ 31

Table S 13. Hydrogen Atom Coordinates (Å×104) and Isotropic Displacement Parameters (Å2×103) for **2-Li**........ 32

Scheme S1. Synthesis of benzylphosphines ligands **1**, **2** and **3**........ 34

4. DFT computations for **1a-Li**........ 34

4.1 Cartesian coordinates........ 34

5. Polymerization reactions........ 36

5.1 NMR spectra of selected polymers before purification to determine conversion values........ 36

Figure S25. ^{1}H NMR spectrum of PCL obtained with catalyst **1-Li** (Table 1, entry 1)........ 36

Figure S26. ^{1}H NMR spectrum of PCL obtained with catalyst **2-Li** (Table 1, entry 2)........ 37

Figure S27. ^{1}H NMR spectrum of PCL obtained with catalyst **2-Li$_2$** (Table 1, entry 3)........ 38

Figure S28. ^{1}H NMR spectrum of PCL obtained with catalyst **3-Li$_3$** (Table 1, entry 4)........ 39

Figure S29. ^{1}H NMR spectrum of PLA obtained with catalyst **2-Li** (Table 2, entry 2)........ 40

5.2 NMR spectra of selected polymers following purification........ 41

Figure S30. ^{1}H NMR spectrum of PCL obtained with catalyst **2-Li** (Table 1, entry 2)........ 41

Figure S31. ^{1}H NMR spectrum of PCL obtained with catalyst **3-Li$_3$** (Table 1, entry 4)........ 42

Figure S32. ^{1}H NMR of PLA obtained with catalyst **2-Li** (Table 2, entry 2)........ 43

5.3 Calculation procedure for determining the isotactic probability of the polylactides from the methine region of their ^{1}H NMR spectra giving rise to the values shown in Table 2........ 44

Figure S 33. Deconvolution of the methine region of the homonuclear-decoupled ^{1}H NMR spectrum of PLA using **2-Li** (Table 2, entry 2)........ 44

5.4 Yields of isolated polymers........ 45

Table S 14. Polymerization of ε-CL by lithium complexes at 25 ℃ including observed conversion and yields of isolated polymers. Modified from Table 1 in main text. Cat = catalyst........ 45

Table S 15. Polymerization of *rac*-LA by lithium complexes at 140 ℃ including yields of isolated polymers. Modified from Table 2 in main text. Cat= catalyst........ 45

1. NMR spectroscopic data for all lithium complexes

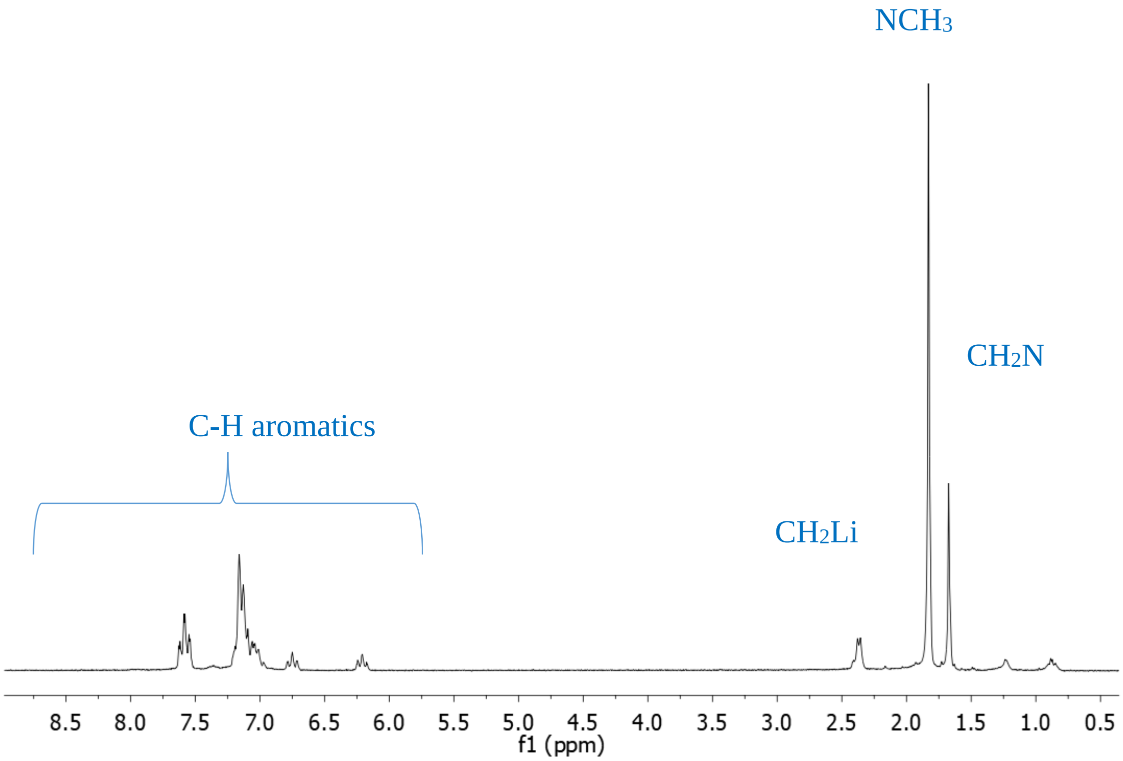

Figure S1. ^{1}H NMR spectrum (400 MHz, C_6D_6, 298 K) of complex **1-Li**.

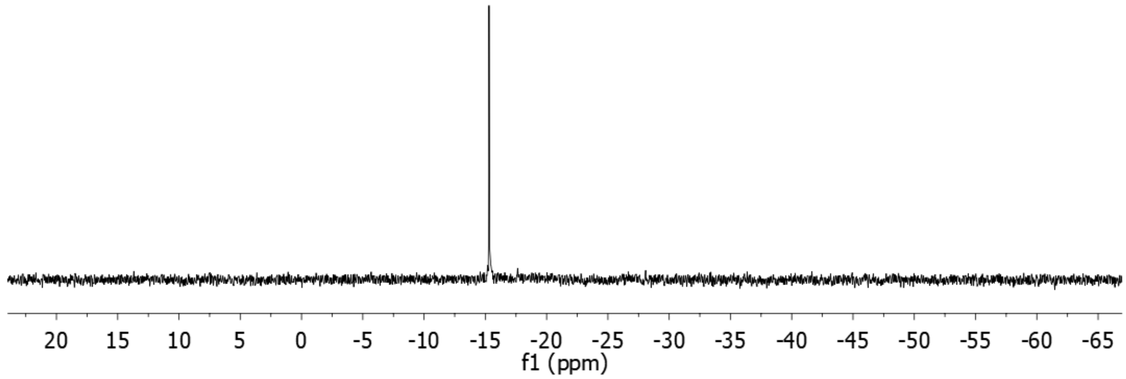

Figure S2. $^{31}P\{^{1}H\}$ NMR spectrum (161.92 MHz, C_6D_6, 298 K) of complex **1-Li**.

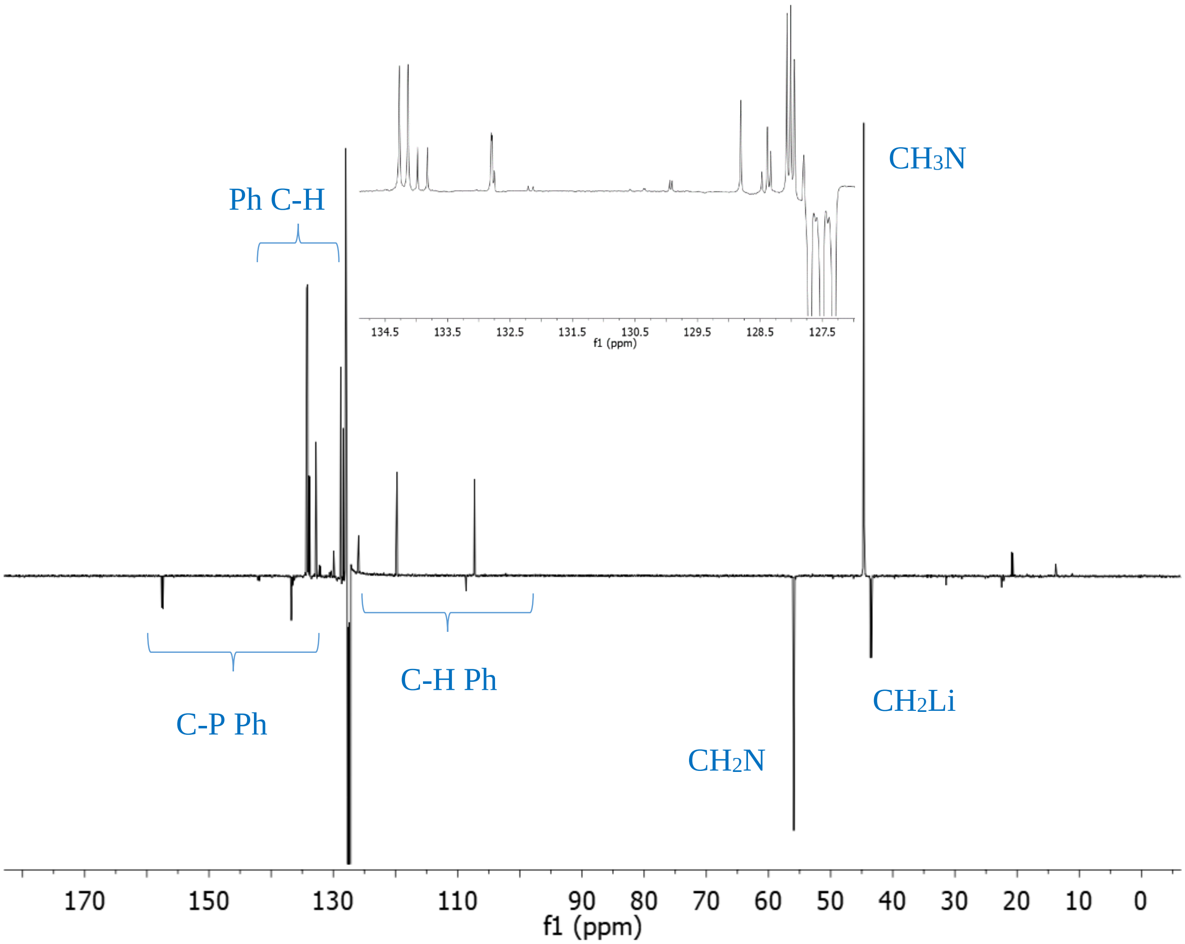

Figure S3. DEPTQ NMR spectrum (100.68 MHz, C_6D_6, 298 K) of complex **1-Li**.

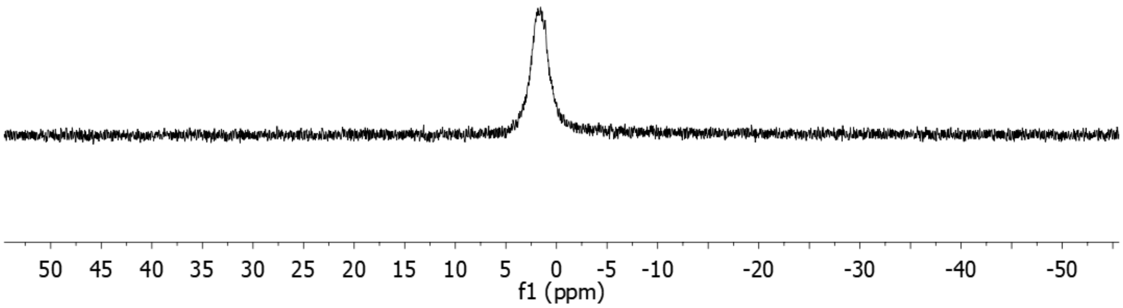

Figure S4. ^{7}Li NMR spectrum (155.45 MHz, C_6D_6, 298 K) of complex **1-Li**.

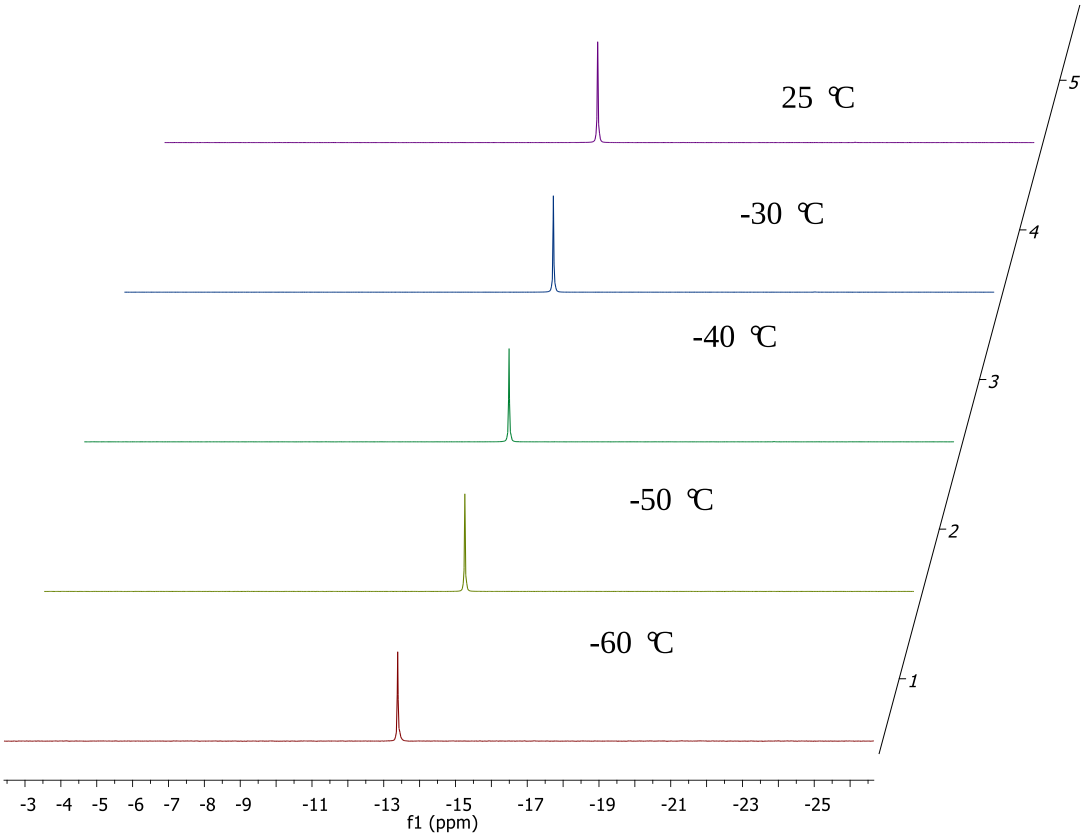

Figure S5. $^{31}P\{^{1}H\}$ NMR spectrum (202.40 MHz, C_7D_8) at variable temperatures of complex **1-Li**.

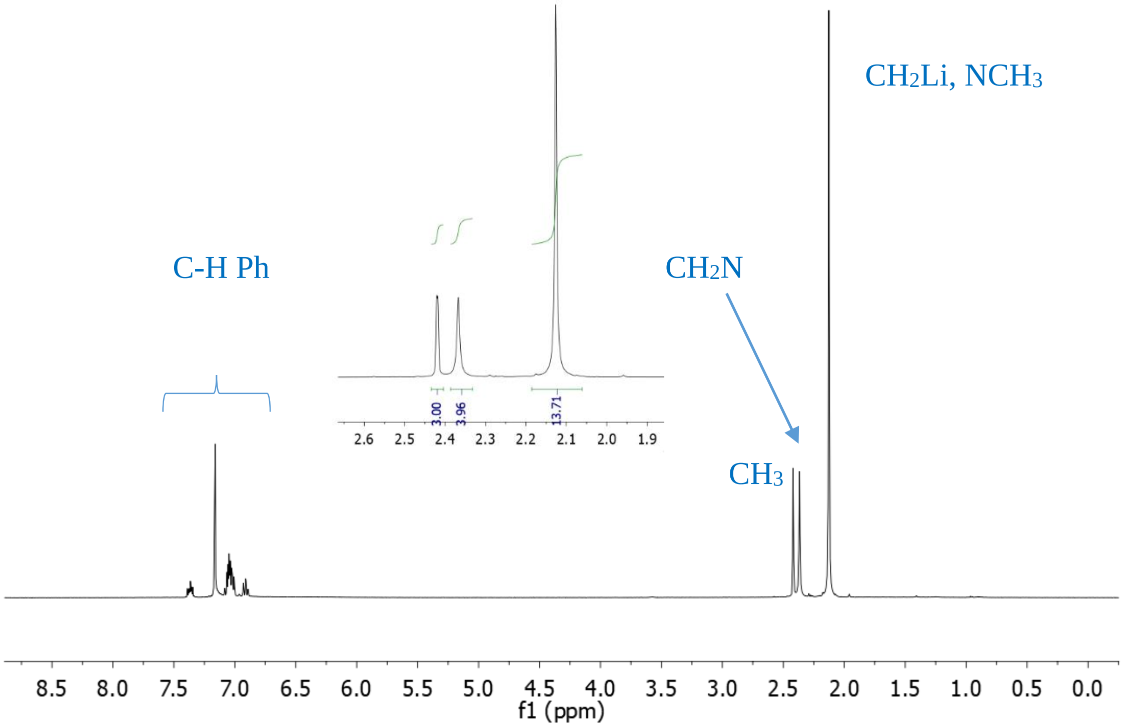

Figure S6. ^{1}H NMR spectrum (700 MHz, C_6D_6, 298 K) of complex **2-Li**.

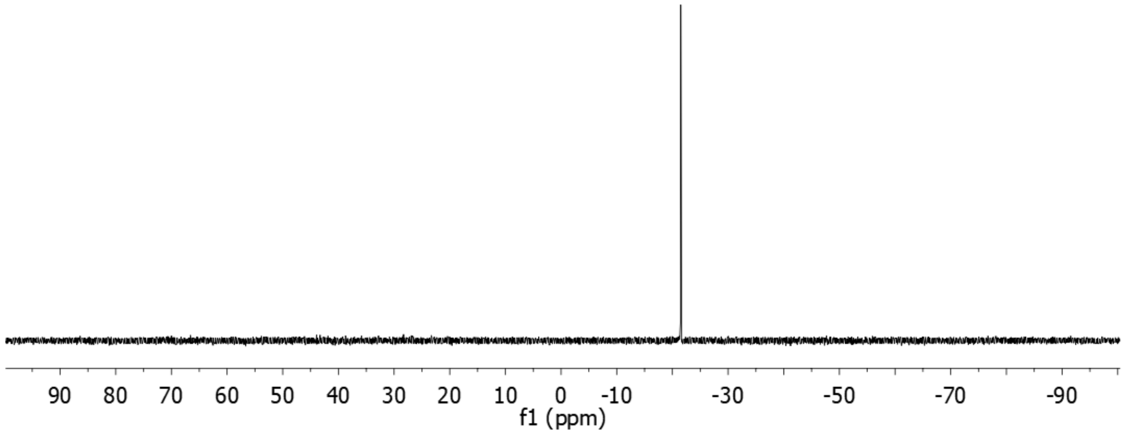

Figure S7. $^{31}P\{^{1}H\}$ NMR spectrum (161.92 MHz, C_6D_6, 298 K) of complex **2-Li**.

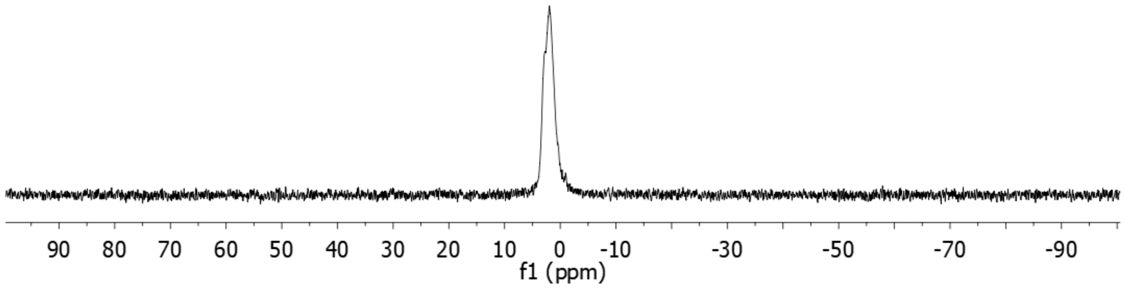

Figure S8. ^{7}Li NMR spectrum (155.45 MHz, C_6D_6, 298 K) of complex **2-Li**.

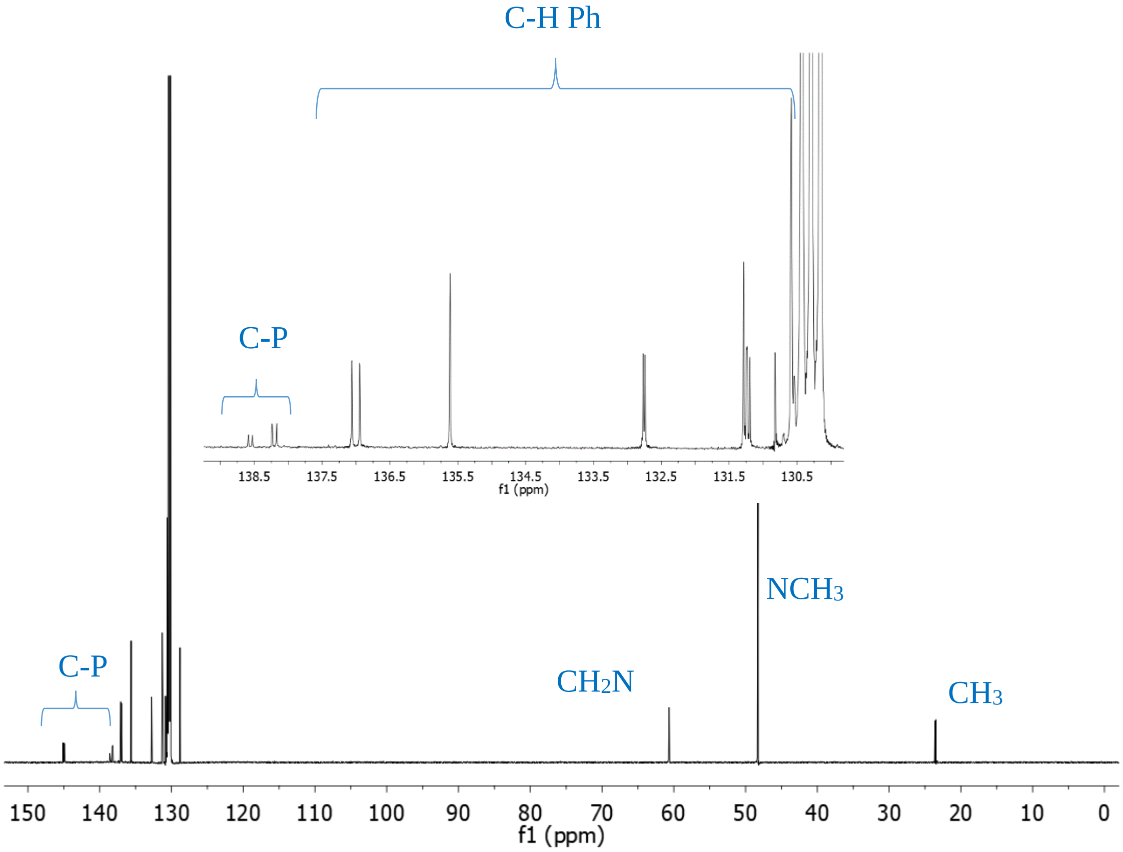

Figure S9. $^{13}C\{^{1}H\}$ NMR spectrum (700 MHz, C_6D_6, 298 K) of complex **2-Li**.

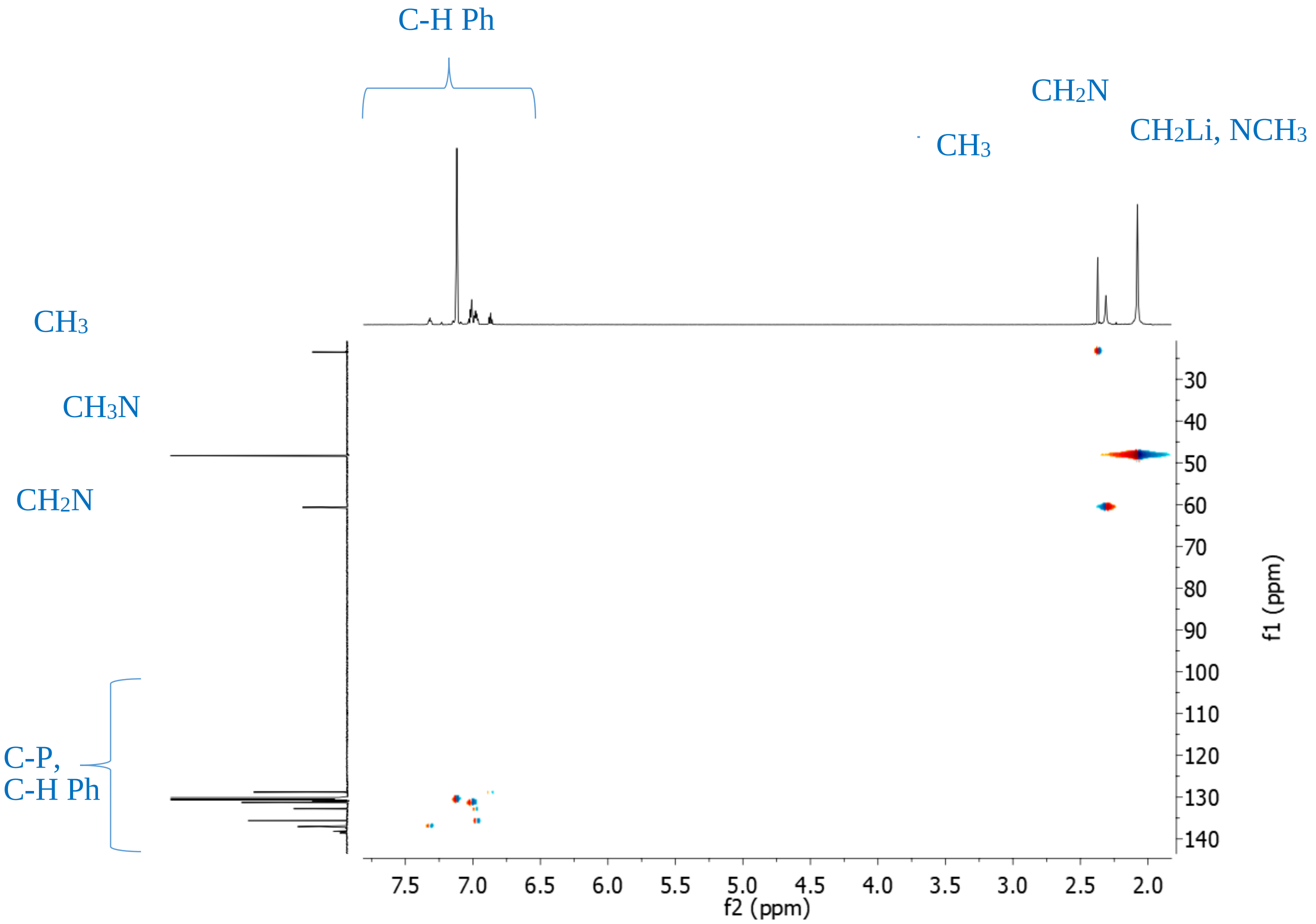

Figure S10. HSQC ^1H-^{13}C NMR spectrum (700 MHz, C_6D_6, 298 K) of complex **2-Li.**

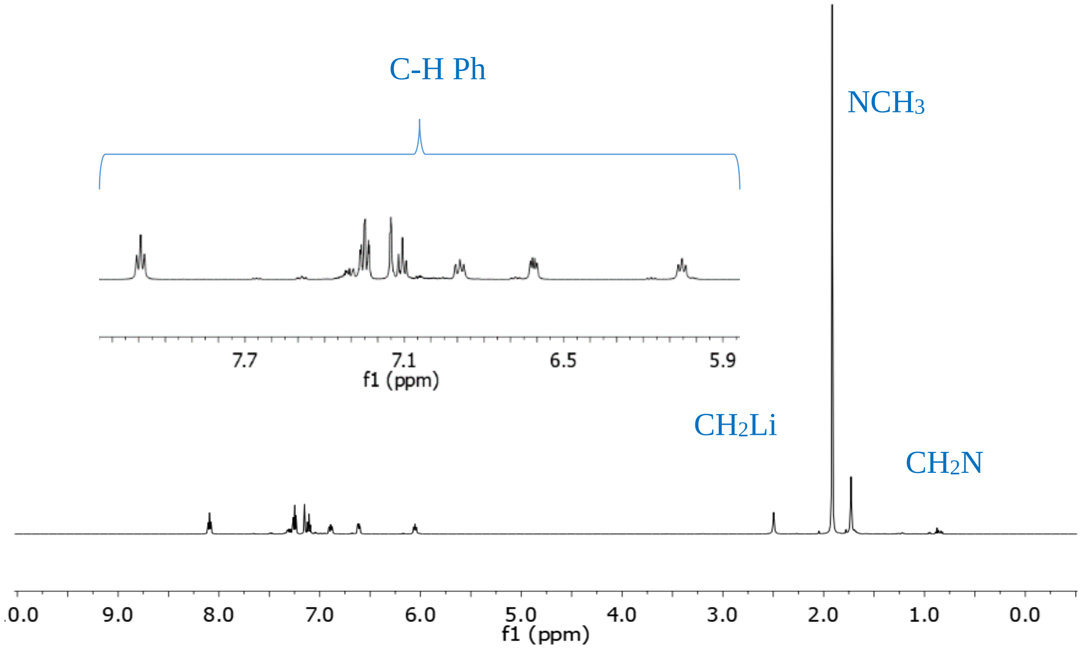

Figure S11. ^1H NMR spectrum (400 MHz, C_6D_6, 298 K) of complex **$2\text{-}Li_2$**.

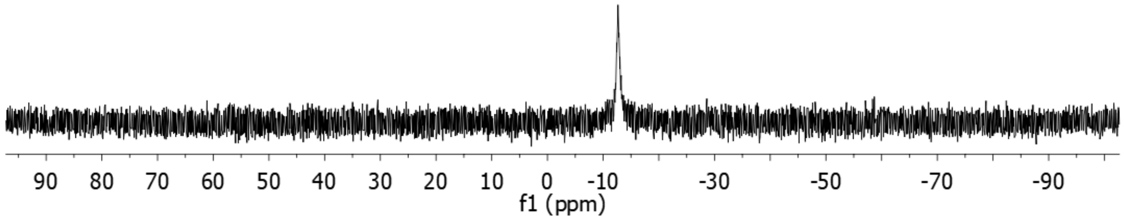

Figure S12. $^{31}P\{^{1}H\}$ NMR spectrum (161.92 MHz, C_6D_6, 298 K) of complex **2-Li$_2$**.

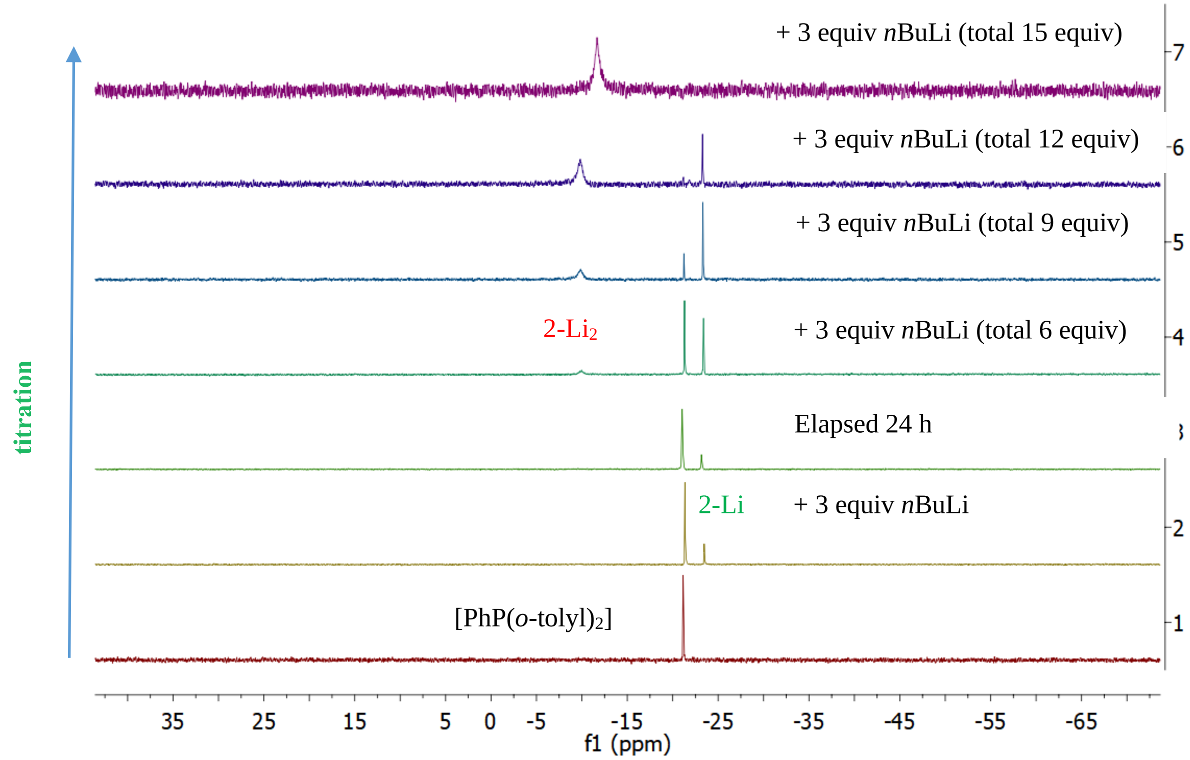

Figure S13. $^{31}P\{^{1}H\}$ NMR spectra for the titration of [PhP(o-tolyl)2] with excess *n*BuLi, evidencing the formation of complexes **2Li** and **2-Li**$_2$ (161.92 MHz, C_6D_6, 25 °C).

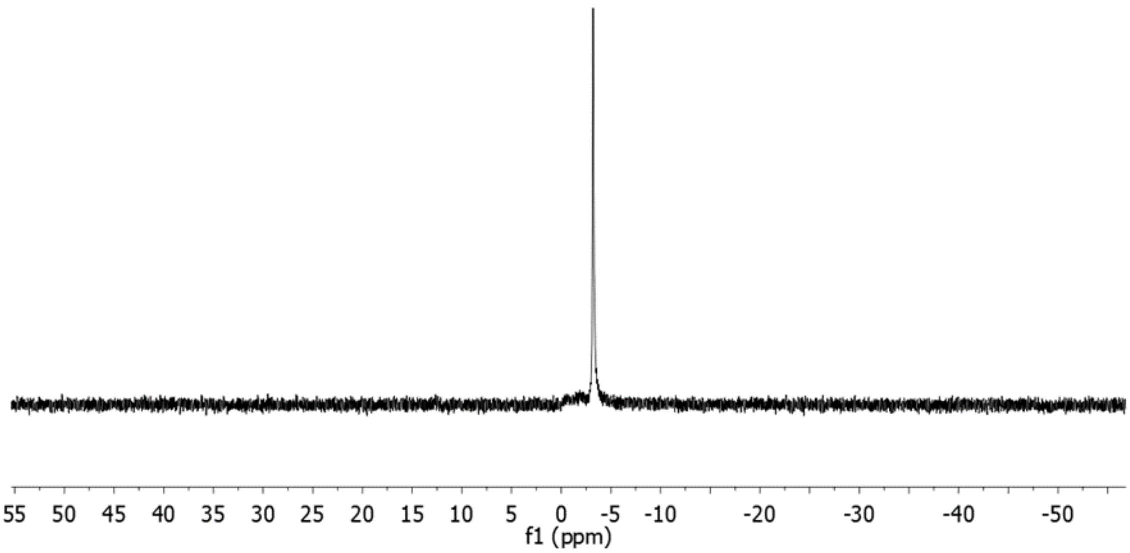

Figure S14. ^{7}Li NMR spectrum (155.45 MHz, C_6D_6, 298 K) of complex **2-Li$_2$**.

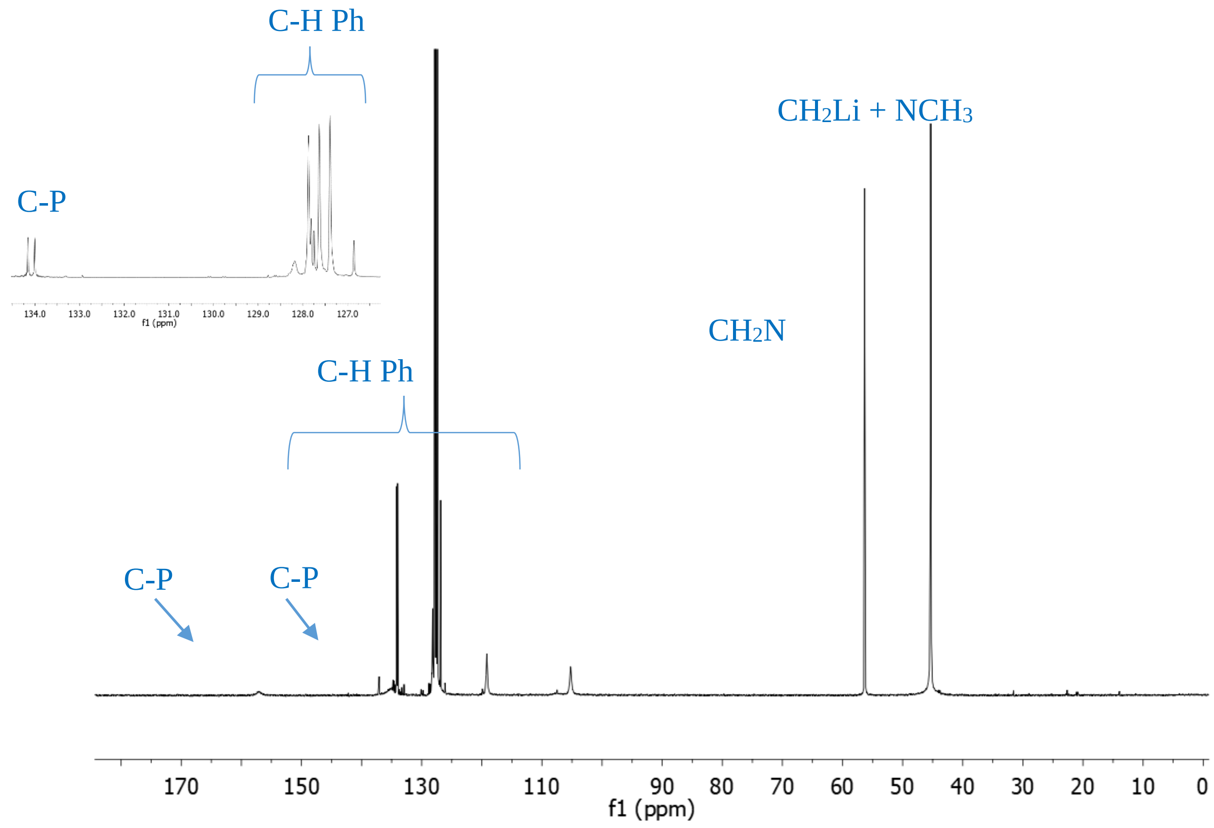

Figure S15. $^{13}C\{^{1}H\}$ NMR spectrum (125.72 MHz, C_6D_6, 25°C) of complex **2-Li$_2$**.

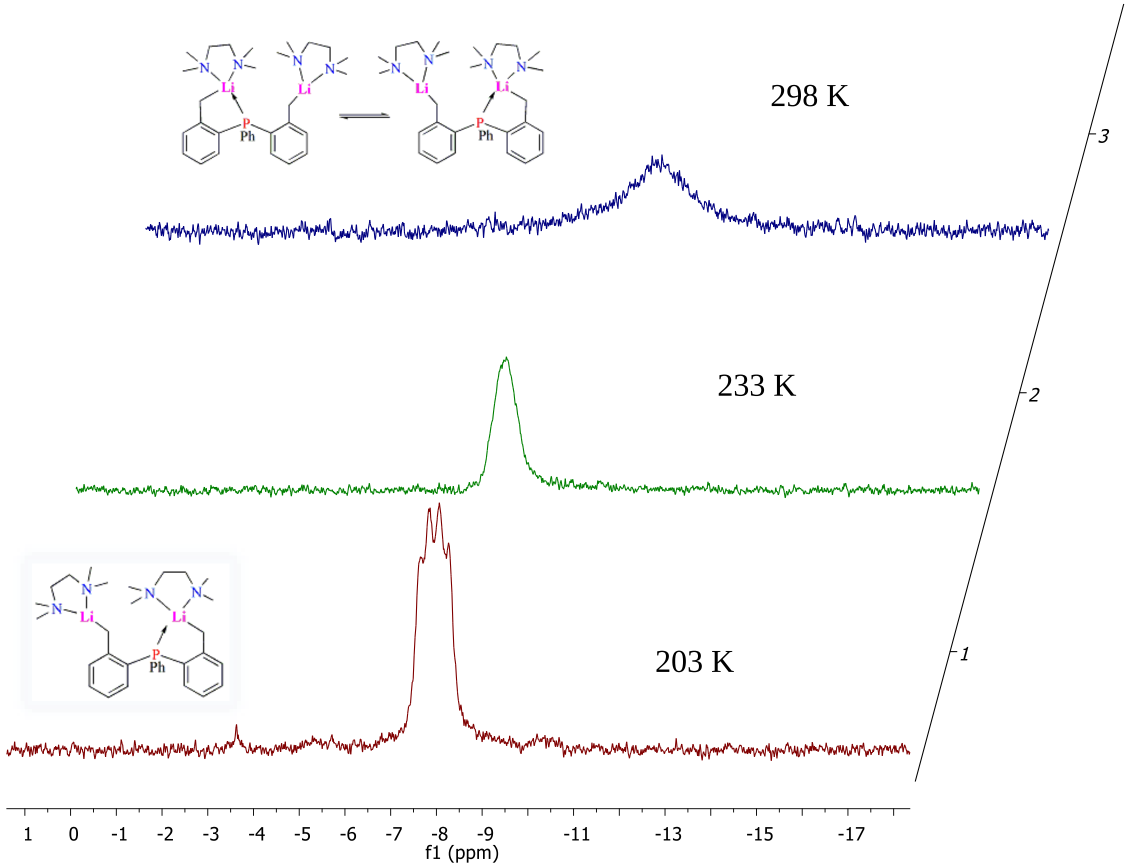

Figure S16. $^{31}P\{^{1}H\}$ NMR spectrum (202.40, toluene-d_8) of complex **2-Li$_2$** at given temperatures.

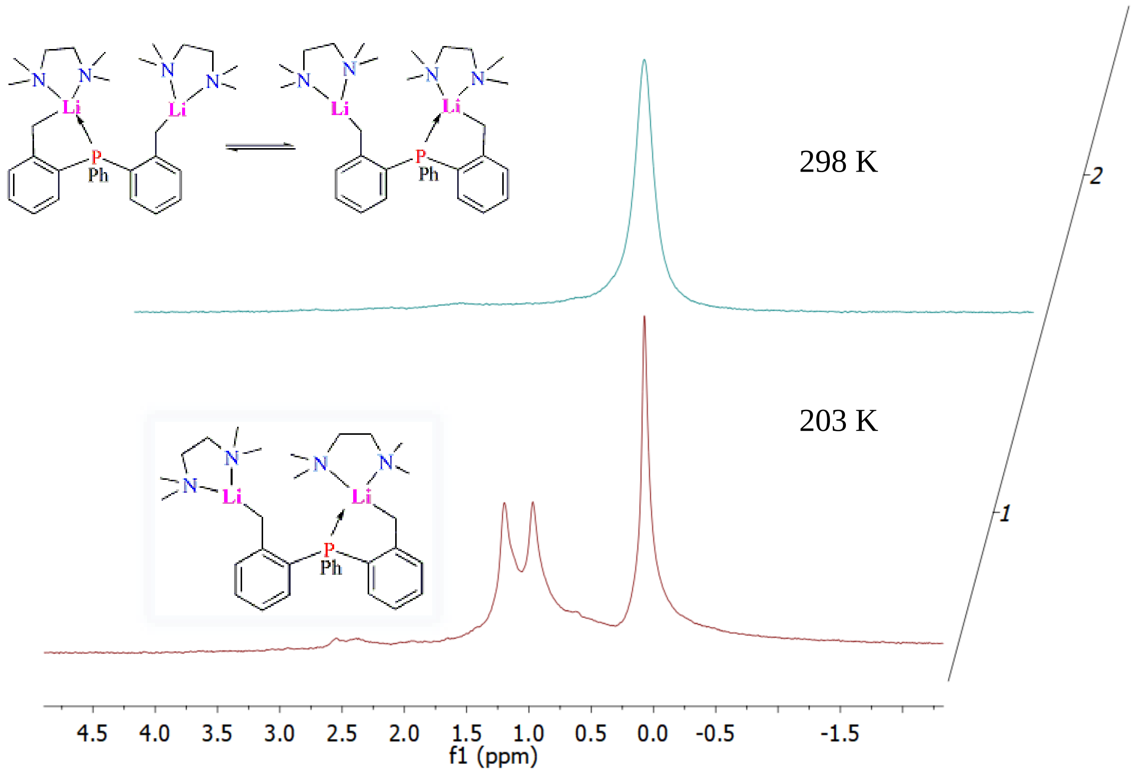

Figure S17. ^{7}Li NMR spectrum at 298 K and 203 K (194.32 MHz, C_7D_8) of complex **2-Li$_2$**

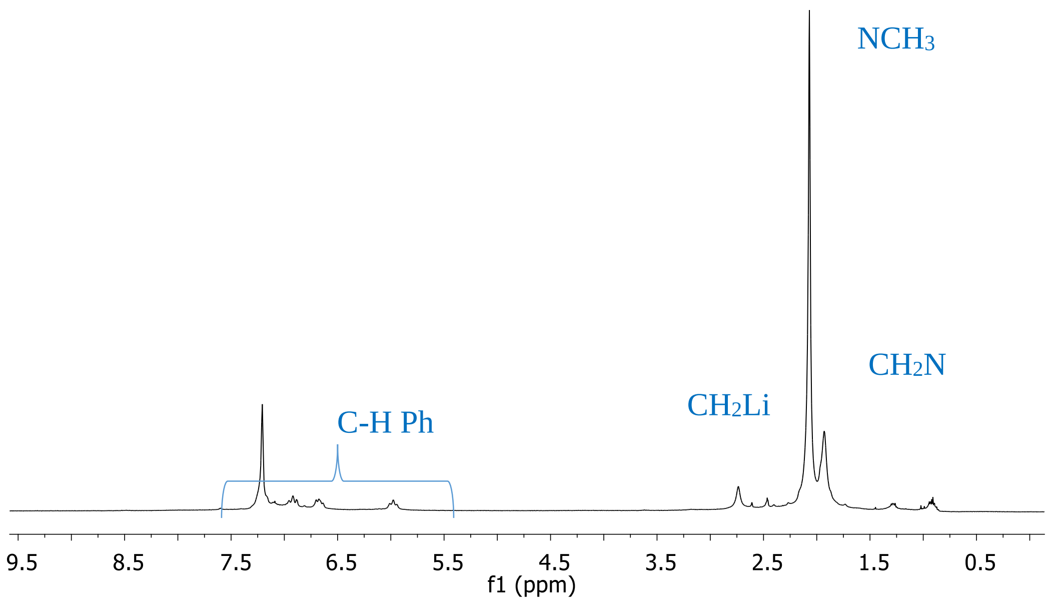

Figure S18. ^{1}H NMR spectrum (400 MHz, C_6D_6, 25 °C) of complex **3-Li$_3$**

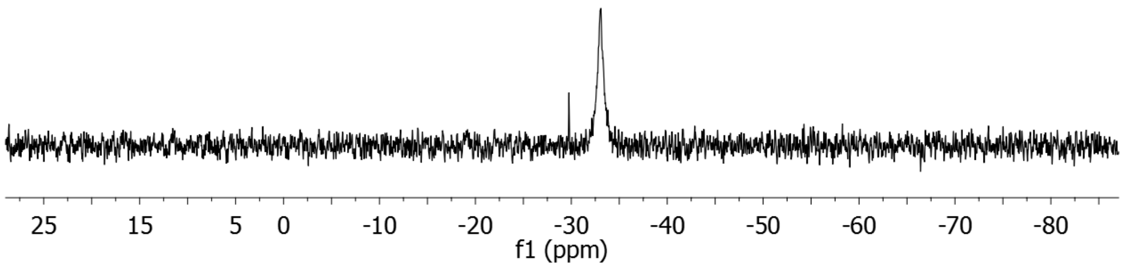

Figure S19. $^{31}P\{^{1}H\}$ NMR spectrum (161.92 MHz, C_6D_6, 298 K) of complex **3-Li$_3$**.

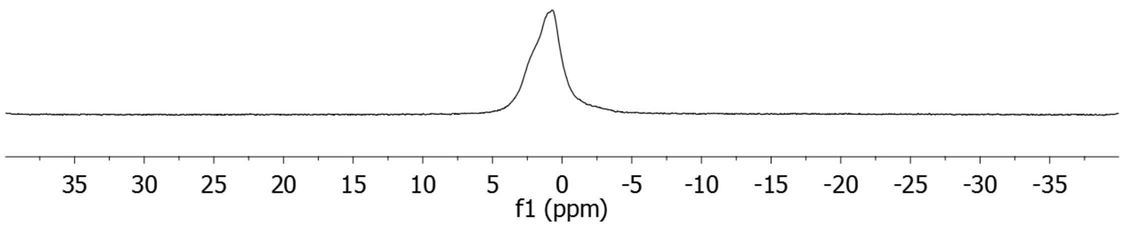

Figure S20. ^{7}Li NMR spectrum (194.32 MHz, C_6D_6, 298 K) of complex **3-Li$_3$**.

2. FT IR spectra

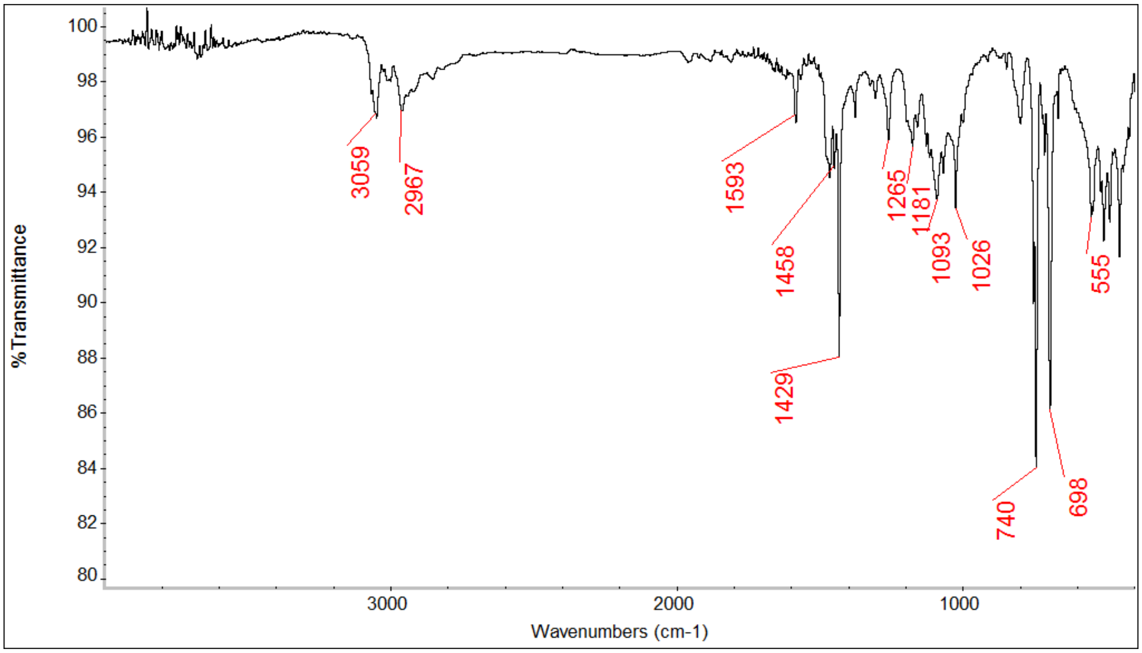

Figure S21. Infrared spectrum of **1-Li** (KBr disc)

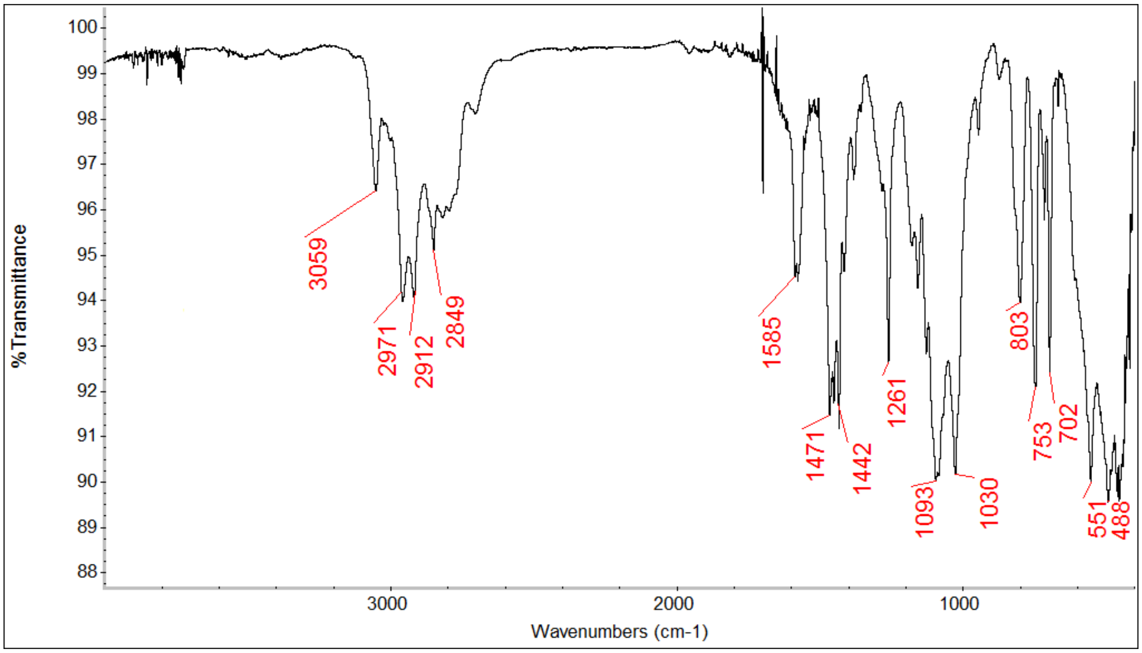

Figure S22. Infrared spectrum of **2-Li** (KBr disc)

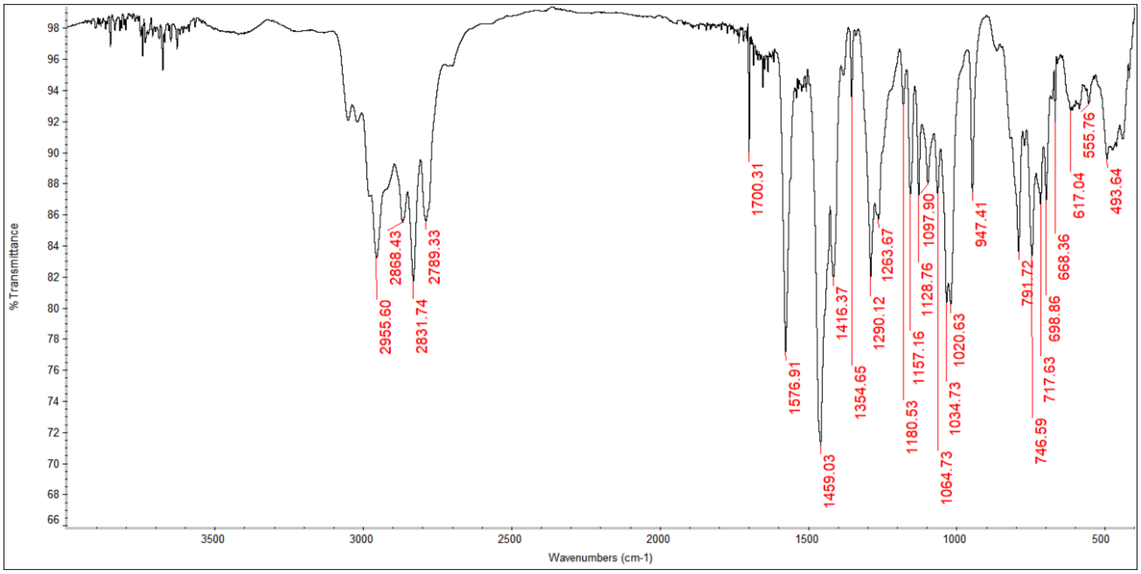

Figure S23. Infrared spectrum of **2-Li$_2$** (KBr disc).

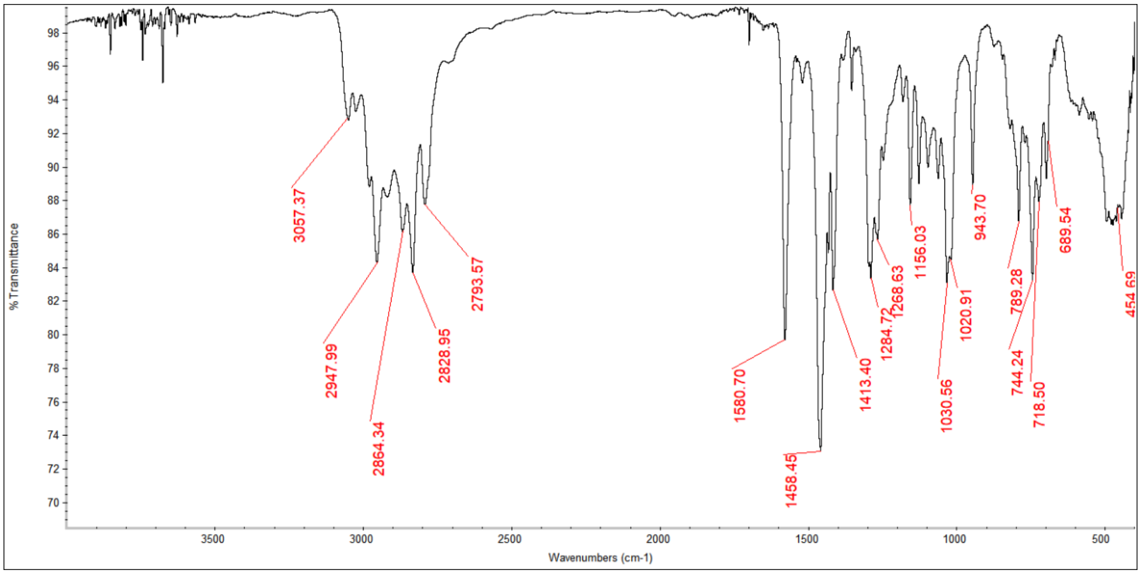

Figure S24. Infrared spectrum of **2-Li$_3$** (KBr disc).

3. X-ray diffraction data

3.1 X-ray diffraction data of complex **1-Li**

Table S 1. Crystal data and structure refinement for **1-Li**.

Identification code	MM89a
Empirical formula	$C_{25}H_{32}LiN_2P$
Formula weight	398.43
Temperature/K	113(18)
Crystal system	monoclinic
Space group	C2/c
a/Å	17.0285(9)
b/Å	14.1666(7)
c/Å	19.4268(10)
α/°	90
β/°	96.613(5)
γ/°	90
Volume/Å3	4655.3(4)
Z	8
ρ_{calc}g/cm3	1.137
μ/mm-1	1.117
F(000)	1712.0
Crystal size/mm3	0.21 × 0.18 × 0.08
Radiation	CuKα (λ = 1.54184)
2Θ range for data collection/°	8.14 to 145.162
Index ranges	−13 ≤ h ≤ 20, −15 ≤ k ≤ 17, −23 ≤ l ≤ 20
Reflections collected	8275
Independent reflections	4529 [R_{int} = 0.0294, R_{sigma} = 0.0353]
Data/restraints/parameters	4529/0/261
Goodness-of-fit on F2	1.047
Final R indexes [I>=2σ (I)]	R_1 = 0.0514, wR_2 = 0.1362
Final R indexes [all data]	R_1 = 0.0566, wR_2 = 0.1424
Largest diff. peak/hole / e Å-3	0.53/-0.43

Table S 2. Fractional Atomic Coordinates (×104) and Equivalent Isotropic Displacement Parameters (Å2×103) for **1-Li**.

Ueq is defined as 1/3 of the trace of the orthogonalised UIJ tensor.

Atom	x	y	z	U(eq)
P1	3265.5(2)	4468.8(3)	6618.6(2)	20.68(14)
C3	1266.4(10)	5659.8(12)	5562.8(9)	26.9(4)
C1	2269.9(9)	4669.2(12)	6205.3(8)	21.1(3)
C8	3839.8(9)	5474.2(12)	6356.7(8)	23.7(3)
N1	1809.5(8)	5389.5(10)	7960.9(7)	25.6(3)
C2	2055(1)	5442.7(12)	5779.5(8)	24.1(3)
C5	875.6(9)	4332.2(12)	6229.2(9)	24.7(3)

C19	4345.9(9)	3043.0(12)	6416.9(8)	23.4(3)
C20	2264.5(11)	5648.2(14)	8628.8(9)	32.1(4)
C17	4335.9(10)	2075.1(12)	5399.7(9)	26.0(3)
C13	4225.7(10)	5483.3(12)	5760.8(9)	26.9(4)
C23	1708.9(11)	6213.4(13)	7500.6(9)	31.1(4)
N2	2975.9(9)	4120.5(12)	8648.5(7)	31.8(3)
C18	4681.6(9)	2336.0(12)	6054.0(9)	26.4(4)
C15	3315.8(9)	3236.9(11)	5469.5(8)	22.3(3)
C16	3652.5(9)	2528.8(12)	5107.1(8)	24.4(3)
C14	3661.3(9)	3509.6(11)	6127.5(8)	21.1(3)
C7	1869.4(9)	3241.1(12)	6843.7(8)	24.7(3)
C6	1677.2(9)	4042.9(11)	6428.9(8)	21.7(3)
C4	683.9(9)	5094.3(12)	5808.2(8)	26.6(4)
C25	3788.9(11)	4456.4(17)	8631.4(10)	43.4(5)
C22	1023.9(10)	5019.8(15)	8066.6(10)	34.7(4)
C11	4727.8(12)	7037.7(15)	6031.2(10)	39.3(5)
C21	2534.6(11)	4782.1(14)	9046.9(9)	33.2(4)
C12	4664.7(11)	6259.2(14)	5602.8(9)	32.3(4)
C10	4342.7(14)	7043.6(15)	6623.2(11)	44.3(5)
C9	3912.9(11)	6265.3(14)	6788(1)	35.4(4)
C24	2991.2(16)	3178.5(16)	8958.3(12)	52.4(6)
Li1	2403.6(17)	4221(2)	7648.2(15)	28.2(6)

Table S 3. Anisotropic Displacement Parameters (Å^2×10^3) for **1-Li**.

The Anisotropic displacement factor exponent takes the form: $-2\pi^2[h^2a^{*2}U_{11}+2hka^*b^*U_{12}+\ldots]$.

Atom	U_{11}	U_{22}	U_{33}	U_{23}	U_{13}	U_{12}
P1	16.8(2)	22.6(2)	23.1(2)	-1.15(14)	4.36(15)	-0.20(13)
C3	26.6(8)	27.8(9)	26.4(8)	2.0(6)	2.7(6)	7.2(7)
C1	18.3(7)	22.9(8)	22.7(7)	-2.9(6)	4.8(5)	1.7(6)
C8	18.8(7)	24.5(8)	27.9(8)	-0.3(6)	3.7(6)	-1.7(6)
N1	20.5(7)	29.0(8)	27.8(7)	-4.6(6)	4.5(5)	-0.4(5)
C2	22.2(8)	25.2(8)	26.1(8)	-1.5(6)	7.2(6)	1.0(6)
C5	18.1(7)	26.2(8)	30.1(8)	-6.7(6)	4.4(6)	-1.2(6)
C19	18.6(7)	25.4(8)	26.3(7)	2.0(6)	3.3(6)	-0.8(6)
C20	30.6(9)	36.2(10)	29.4(9)	-11.3(7)	2.5(7)	2.3(7)
C17	23.7(8)	23.6(8)	32.8(8)	0.9(6)	12.1(6)	0.9(6)
C13	24.2(8)	27.6(9)	29.2(8)	-3.1(6)	4.8(6)	-3.4(6)
C23	30.3(9)	28.8(9)	34.7(9)	-3.8(7)	6.0(7)	5.3(7)
N2	31.5(8)	34.2(9)	29.0(7)	-1.8(6)	0.8(6)	3.7(6)
C18	18.5(7)	26.1(9)	35.2(8)	4.5(7)	5.5(6)	2.7(6)
C15	16.1(7)	24.1(8)	27.3(7)	0.5(6)	4.5(5)	0.1(6)
C16	22.5(8)	25.9(8)	25.9(7)	-2.1(6)	6.8(6)	-3.2(6)
C14	16.0(7)	21.6(8)	26.9(7)	0.8(6)	6.8(5)	-1.1(6)
C7	21.0(7)	24.0(8)	29.7(8)	-2.0(6)	6.0(6)	-1.0(6)

C6	20.3(7)	21.9(8)	23.4(7)	-6.2(6)	4.6(5)	-0.2(6)
C4	20.1(7)	30.0(9)	29.3(8)	-6.2(7)	0.6(6)	5.7(6)
C25	26.6(10)	67.0(16)	35.9(10)	-1.3(9)	0.3(8)	8.4(9)
C22	21.6(8)	41.6(11)	42.3(10)	-7.1(8)	10.1(7)	-1.1(7)
C11	45.0(11)	34(1)	40.4(10)	-3.3(8)	11.4(8)	-18.4(9)
C21	32.0(9)	43.0(11)	25.2(8)	-4.0(7)	4.9(7)	0.7(8)
C12	31.9(9)	35.3(10)	31.3(8)	-1.9(7)	10.9(7)	-8.5(7)
C10	57.9(13)	34.2(11)	43.6(11)	-13.7(9)	17.6(9)	-16.2(9)
C9	39.9(10)	33.9(10)	34.7(9)	-7.3(7)	14.2(7)	-8.7(8)
C24	66.1(15)	40.0(12)	47.2(12)	5.0(9)	-11.0(11)	3.9(11)

Table S 4. Bond Lengths for **1-Li**.

Atom	Atom	Length/Å	Atom	Atom	Length/Å
P1	C1	1.8128(16)	C19	C14	1.400(2)
P1	C8	1.8325(17)	C20	C21	1.513(3)
P1	C14	1.8328(16)	C17	C18	1.388(2)
P1	Li1	2.637(3)	C17	C16	1.392(2)
C3	C2	1.395(2)	C13	C12	1.383(2)
C3	C4	1.401(3)	N2	C25	1.468(3)
C1	C2	1.396(2)	N2	C21	1.475(2)
C1	C6	1.447(2)	N2	C24	1.463(3)
C8	C13	1.395(2)	N2	Li1	2.076(3)
C8	C9	1.396(2)	C15	C16	1.387(2)
N1	C20	1.479(2)	C15	C14	1.399(2)
N1	C23	1.468(2)	C7	C6	1.409(2)
N1	C22	1.473(2)	C7	Li1	2.206(3)
N1	Li1	2.068(3)	C6	Li1	2.555(3)
C5	C6	1.435(2)	C11	C12	1.378(3)
C5	C4	1.371(2)	C11	C10	1.388(3)
C19	C18	1.386(2)	C10	C9	1.381(3)

Table S 5. Bond Angles for **1-Li**.

Atom	Atom	Atom	Angle/˚	Atom	Atom	Atom	Angle/˚
C1	P1	C8	104.90(7)	C24	N2	Li1	115.58(15)
C1	P1	C14	105.48(7)	C19	C18	C17	120.14(15)
C1	P1	Li1	77.46(8)	C16	C15	C14	120.58(14)
C8	P1	C14	101.06(7)	C15	C16	C17	120.11(15)
C8	P1	Li1	132.50(9)	C19	C14	P1	118.69(12)
C14	P1	Li1	124.48(8)	C15	C14	P1	122.64(12)
C2	C3	C4	117.79(15)	C15	C14	C19	118.66(14)
C2	C1	P1	123.59(12)	C6	C7	Li1	87.00(12)
C2	C1	C6	120.97(14)	C1	C6	Li1	86.44(11)
C6	C1	P1	114.83(12)	C5	C6	C1	114.72(14)
C13	C8	P1	123.71(13)	C5	C6	Li1	124.49(12)
C13	C8	C9	118.18(15)	C7	C6	C1	122.80(14)

C9	C8	P1	118.08(13)	C7	C6	C5	122.39(15)
C20	N1	Li1	103.40(13)	C7	C6	Li1	59.57(11)
C23	N1	C20	110.48(14)	C5	C4	C3	121.61(15)
C23	N1	C22	108.87(14)	C12	C11	C10	119.58(18)
C23	N1	Li1	118.96(13)	N2	C21	C20	112.04(14)
C22	N1	C20	111.00(14)	C11	C12	C13	120.55(17)
C22	N1	Li1	103.84(14)	C9	C10	C11	120.05(18)
C3	C2	C1	121.97(15)	C10	C9	C8	120.98(17)
C4	C5	C6	122.67(15)	N1	Li1	P1	116.74(13)
C18	C19	C14	120.67(15)	N1	Li1	N2	88.42(12)
N1	C20	C21	111.48(15)	N1	Li1	C7	122.41(15)
C18	C17	C16	119.83(15)	N1	Li1	C6	98.64(12)
C12	C13	C8	120.64(16)	N2	Li1	P1	118.48(13)
C25	N2	C21	110.16(15)	N2	Li1	C7	136.95(16)
C25	N2	Li1	107.39(14)	N2	Li1	C6	170.37(16)
C21	N2	Li1	103.60(13)	C7	Li1	P1	76.51(10)
C24	N2	C25	109.45(17)	C7	Li1	C6	33.42(7)
C24	N2	C21	110.47(17)	C6	Li1	P1	64.04(7)

Table S 6. Torsion Angles for MM89a.

A	B	C	D	Angle/˚	A	B	C	D	Angle/˚
P1	C1	C2	C3	-169.24(13)	C14	P1	C1	C6	83.47(12)
P1	C1	C6	C5	166.25(11)	C14	P1	C8	C13	19.87(15)
P1	C1	C6	C7	-10.3(2)	C14	P1	C8	C9	-158.29(14)
P1	C1	C6	Li1	39.66(12)	C14	C19	C18	C17	-0.7(2)
P1	C8	C13	C12	-178.49(14)	C14	C15	C16	C17	0.6(2)
P1	C8	C9	C10	179.50(17)	C6	C1	C2	C3	1.4(2)
C1	P1	C8	C13	-89.60(15)	C6	C5	C4	C3	-2.0(2)
C1	P1	C8	C9	92.24(15)	C4	C3	C2	C1	2.4(2)
C1	P1	C14	C19	-164.97(12)	C4	C5	C6	C1	5.5(2)
C1	P1	C14	C15	16.30(15)	C4	C5	C6	C7	-177.93(15)
C8	P1	C1	C2	0.85(15)	C4	C5	C6	Li1	109.03(18)
C8	P1	C1	C6	-170.30(11)	C25	N2	C21	C20	78.39(18)
C8	P1	C14	C19	86.01(13)	C22	N1	C20	C21	71.93(19)
C8	P1	C14	C15	-92.71(14)	C11	C10	C9	C8	-1.7(3)
C8	C13	C12	C11	-0.1(3)	C12	C11	C10	C9	1.3(3)
N1	C20	C21	N2	53.9(2)	C10	C11	C12	C13	-0.4(3)
C2	C3	C4	C5	-2.1(2)	C9	C8	C13	C12	-0.3(3)
C2	C1	C6	C5	-5.1(2)	C24	N2	C21	C20	-160.58(16)

C2 C1 C6 C7 178.33(14) Li1 P1 C1 C2 131.92(15)
C2 C1 C6 Li1 -131.73
(15) Li1 P1 C1 C6 -39.22(12)
C13 C8 C9 C10 1.2(3) Li1 P1 C8 C13 -176.11
(14)
C23 N1 C20 C21 -167.19
(14) Li1 P1 C8 C9 5.7(2)
C18 C19 C14 P1 -177.73
(12) Li1 P1 C14 C19 -79.73(15)
C18 C19 C14 C15 1.1(2) Li1 P1 C14 C15 101.54(15)
C18 C17 C16 C15 -0.3(2) Li1 N1 C20 C21 -38.85(18)
C16 C17 C18 C19 0.3(2) Li1 N2 C21 C20 -36.22(19)
C16 C15 C14 P1 177.71(12) Li1 C7 C6 C1 62.36(17)
C16 C15 C14 C19 -1.0(2) Li1 C7 C6 C5 -113.90
(16)
C14 P1 C1 C2 -105.39
(14)

Crystal structure determination of [MM89a]

Crystal Data for $C_{25}H_{32}LiN_2P$ (M =398.43 g/mol): monoclinic, space group C2/c (no. 15), a = 17.0285(9) Å, b = 14.1666(7) Å, c = 19.4268(10) Å, β = 96.613(5)°, V = 4655.3(4) $Å^3$, Z = 8, T = 113(18) K, μ(CuKα) = 1.117 mm^{-1},

Table S 7. Hydrogen Atom Coordinates (Å×10^4) and Isotropic Displacement Parameters ($Å^2$×10^3) for **1-Li**.

Atom	x	y	z	U(eq)
H3	1132	6163	5265	32
H2	2450	5825	5636	29
H5	470	3989	6392	30
H19	4577	3209	6857	28
H20A	1938	6035	8894	39
H20B	2722	6018	8542	39
H17	4560	1598	5157	31
H13	4188	4963	5467	32
H23A	1418	6693	7711	47
H23B	1424	6031	7066	47
H23C	2218	6455	7424	47
H18	5140	2036	6249	32
H15	2856	3533	5273	27
H16	3421	2357	4668	29
H7A	2245	2819	6663	30
H7B	1414	2899	6972	30
H4	154	5239	5682	32
H25A	3779	5084	8445	65
H25B	4060	4045	8345	65
H25C	4060	4460	9093	65
H22A	1084	4479	8366	52
H22B	746	4840	7628	52

H22C 730 5499 8274 52
H11 5026 7556 5924 47
H21A 2869 4976 9461 40
H21B 2077 4462 9191 40
H12 4919 6255 5204 39
H10 4374 7572 6909 53
H9 3669 6268 7192 43
H24A 3276 2755 8691 79
H24B 2460 2954 8963 79
H24C 3248 3208 9424 79

D_{calc} = 1.137 g/cm^3, 8275 reflections measured (8.14° ≤ 2Θ ≤ 145.162°), 4529 unique (R_{int} = 0.0294, R_{sigma} = 0.0353) which were used in all calculations. The final R_1 was 0.0514 (I > 2σ(I)) and wR_2 was 0.1424 (all data).

Refinement model description

Number of restraints - 0, number of constraints - unknown.

Details:

1. Fixed Uiso
At 1.2 times of:
All C(H) groups, All C(H,H) groups
At 1.5 times of:
All C(H,H,H) groups
2.a Secondary CH2 refined with riding coordinates:
C20(H20A,H20B), C7(H7A,H7B), C21(H21A,H21B)
2.b Aromatic/amide H refined with riding coordinates:
C3(H3), C2(H2), C5(H5), C19(H19), C17(H17), C13(H13), C18(H18), C15(H15), C16(H16), C4(H4), C11(H11), C12(H12), C10(H10), C9(H9)
2.c Idealised Me refined as rotating group:
C23(H23A,H23B,H23C), C25(H25A,H25B,H25C), C22(H22A,H22B,H22C), C24(H24A,H24B, H24C)

3.2 X-ray diffraction data of complex **2-Li**

Table S 8. Crystal data and structure refinement for 2-Li.

Identification code	MM122
Empirical formula	$C_{26}H_{34}LiN_2P$
Formula weight	412.46
Temperature/K	100.0(3)
Crystal system	monoclinic
Space group	$P2_1/n$
a/Å	10.90505(11)
b/Å	18.17218(18)
c/Å	12.49103(13)
α/°	90
β/°	103.7696(10)
γ/°	90
Volume/$Å^3$	2404.19(4)
Z	4
$\rho_{calc}g/cm^3$	1.140
μ/mm^{-1}	1.097
F(000)	888.0
Crystal size/mm^3	0.21 × 0.16 × 0.12
Radiation	CuKα (λ = 1.54184)
2Θ range for data collection/°	8.764 to 145.202
Index ranges	$-12 \le h \le 13$, $-22 \le k \le 18$, $-15 \le l \le 11$
Reflections collected	8793
Independent reflections	4677 [R_{int} = 0.0109, R_{sigma} = 0.0153]
Data/restraints/parameters	4677/0/282
Goodness-of-fit on F^2	1.042
Final R indexes [I>=2σ (I)]	R_1 = 0.0685, wR_2 = 0.1834
Final R indexes [all data]	R_1 = 0.0704, wR_2 = 0.1851
Largest diff. peak/hole / e $Å^{-3}$	1.70/-0.64

MM122

Table S 9. Fractional Atomic Coordinates (×10^4) and Equivalent Isotropic Displacement Parameters ($Å^2$×10^3) for MM122.

U_{eq} is defined as 1/3 of the trace of the orthogonalised U_{IJ} tensor.

Atom	x	y	z	U(eq)
P7	1904.8(5)	1260.4(3)	7728.4(5)	19.44(19)
C1	2642(2)	1248.7(13)	9218.2(19)	21.8(5)
C4	3803(3)	1245.2(18)	11461(2)	38.7(7)
C9	-714(2)	1220.3(13)	6981(2)	25.4(5)
C20	4528(2)	1328.8(13)	7310(2)	23.0(5)
C8	319(2)	922.3(12)	7732.6(18)	20.2(5)

N26	1998(2)	1601.0(12)	4581.7(17)	29.4(5)
N23	2786(2)	2975.3(12)	5686.6(19)	32.9(5)
C19	3872(2)	670.8(13)	6964.7(18)	20.6(5)
C2	2395(2)	1824.6(13)	9891(2)	27.1(5)
C12	-1110(2)	145.6(13)	8438.0(19)	24.8(5)
C18	4343(2)	127.9(13)	6333.7(19)	23.6(5)
C6	3448(2)	676.2(15)	9690(2)	29.2(5)
C3	2987(3)	1813.1(16)	11007(2)	34.6(6)
C13	104(2)	385.2(13)	8465.2(19)	23.0(5)
C10	-1931(2)	983.2(15)	6960(2)	29.5(5)
C15	2013(2)	-143.2(14)	6773(2)	28.5(5)
C17	3697(2)	-505.6(14)	5965(2)	27.7(5)
Li30	2918(4)	1887(2)	6193(3)	25.3(8)
C11	-2130(2)	448.6(14)	7688(2)	26.3(5)
C14	2645(2)	509.0(13)	7163.2(18)	21.9(5)
C21	1487(3)	2435.8(15)	9418(2)	39.4(7)
C5	4021(3)	670.3(18)	10808(2)	37.1(6)
C16	2522(2)	-654.9(15)	6182(2)	32.4(6)
C24	2371(4)	2918.9(19)	4474(3)	57.6(10)
C25	1567(4)	2287.9(18)	4062(3)	54.2(9)
C27	3008(4)	1300(2)	4098(3)	56.1(10)
C28	1035(4)	1016(2)	4402(3)	57.9(10)
C22	1736(4)	3310(2)	6066(4)	70.3(12)
C29	3929(4)	3397(2)	5974(4)	69.4(12)

Table S 10. Anisotropic Displacement Parameters (Å^2×10^3) for **2-Li**.

The Anisotropic displacement factor exponent takes the form: $-2\pi^2[h^2a^{*2}U_{11}+2hka^*b^*U_{12}+\ldots]$.

Atom	U_{11}	U_{22}	U_{33}	U_{23}	U_{13}	U_{12}
P7	19.0(3)	20.5(3)	19.5(3)	0.2(2)	6.0(2)	0.4(2)
C1	21.1(11)	24.9(12)	20.9(11)	-0.6(8)	7.8(9)	-3.4(9)
C4	33.3(14)	61.2(19)	21.1(12)	0.0(12)	5.8(11)	-5.6(13)
C9	24.9(12)	29.6(12)	22.3(11)	5.5(9)	6.7(9)	4.1(9)
C20	17.3(11)	26.9(12)	24.2(12)	-0.9(9)	4.1(9)	0.8(9)
C8	20.4(10)	21.8(11)	19.4(10)	-2.1(8)	6.7(8)	1.6(8)
N26	30.5(11)	36.9(12)	20.4(10)	-1.0(8)	4.9(8)	-8.0(9)
N23	40.9(13)	23.7(10)	33.2(12)	2.1(9)	7.3(10)	3.4(9)
C19	18.4(10)	25.2(11)	17.2(10)	2.3(8)	2.3(8)	3.1(9)
C2	32.5(13)	23.6(12)	27.1(12)	-2.8(9)	11(1)	-6.2(10)
C12	27.3(12)	25.3(12)	23.4(11)	0.1(9)	8.9(9)	-2.5(9)
C18	17.9(10)	31.5(12)	21.5(11)	0.0(9)	4.9(9)	3.8(9)
C6	24.8(12)	38.2(14)	26.0(12)	1.5(10)	8.5(10)	6.4(10)
C3	41.8(15)	37.7(14)	26.4(13)	-7.5(11)	12.4(11)	-9.6(12)
C13	21.8(11)	24.3(11)	22.0(11)	2.0(9)	3.2(9)	2.2(9)
C10	21.1(11)	37.9(14)	27.8(12)	4.4(11)	2.5(9)	4.8(10)
C15	24.3(12)	29.0(13)	34.5(13)	-7.6(10)	12(1)	-3.8(10)

C17	26.6(12)	30.4(13)	26.8(12)	-6.5(10)	7.5(10)	5(1)
Li30	25.5(19)	28(2)	21.6(19)	0.4(16)	3.5(15)	1.5(16)
C11	20.6(11)	31.8(13)	27.3(12)	-3.8(10)	7.3(9)	-2.1(9)
C14	20.6(11)	24.6(11)	20.8(11)	-1.6(9)	5.6(8)	0.8(9)
C21	59.2(19)	24.7(13)	36.6(15)	-2.1(11)	16.2(13)	6.1(12)
C5	27.5(13)	55.2(18)	28.6(13)	10.4(12)	6.9(11)	9.4(12)
C16	29.9(13)	28.7(13)	40.1(14)	-12.3(11)	11.4(11)	-5.2(10)
C24	71(2)	44.4(18)	43.9(18)	21.4(15)	-12.1(16)	-15.6(17)
C25	77(2)	46.5(18)	28.0(14)	0.4(13)	-9.7(15)	23.2(17)
C27	79(3)	56(2)	35.3(17)	-1.4(14)	18.0(17)	21.5(18)
C28	60(2)	67(2)	42.7(18)	-0.4(17)	2.8(16)	-23.6(18)
C22	79(3)	49(2)	88(3)	-4(2)	30(2)	20(2)
C29	71(3)	52(2)	70(3)	9.3(19)	-12(2)	-30.0(19)

Table S 11. Bond Lengths for **2-Li**.

Atom	Atom	Length/Å	Atom	Atom	Length/Å
P7	C1	1.843(2)	N23	C24	1.477(4)
P7	C8	1.836(2)	N23	C22	1.471(4)
P7	Li30	2.684(4)	N23	C29	1.434(4)
P7	C14	1.813(2)	C19	C18	1.432(3)
C1	C2	1.408(3)	C19	Li30	2.533(5)
C1	C6	1.399(3)	C19	C14	1.448(3)
C4	C3	1.392(4)	C2	C3	1.391(4)
C4	C5	1.381(4)	C2	C21	1.511(4)
C9	C8	1.393(3)	C12	C13	1.386(3)
C9	C10	1.389(3)	C12	C11	1.387(3)
C20	C19	1.407(3)	C18	C17	1.371(4)
C20	Li30	2.211(5)	C6	C5	1.388(4)
C8	C13	1.395(3)	C10	C11	1.383(4)
N26	Li30	2.090(4)	C15	C14	1.399(3)
N26	C25	1.434(4)	C15	C16	1.383(4)
N26	C27	1.481(4)	C17	C16	1.398(4)
N26	C28	1.473(4)	C24	C25	1.461(5)
N23	Li30	2.071(5)			

Table S 12. Bond Angles for **2-Li**.

Atom	Atom	Atom	Angle/˚	Atom	Atom	Atom	Angle/˚
C1	P7	Li30	124.86(11)	C18	C19	C14	114.9(2)
C8	P7	C1	100.25(10)	C14	C19	Li30	85.97(16)
C8	P7	Li30	133.79(11)	C1	C2	C21	120.9(2)
C14	P7	C1	104.89(11)	C3	C2	C1	118.5(2)
C14	P7	C8	104.84(10)	C3	C2	C21	120.5(2)
C14	P7	Li30	75.16(12)	C13	C12	C11	120.0(2)
C2	C1	P7	119.63(18)	C17	C18	C19	122.7(2)

C6	C1	P7	120.87(18)	C5	C6	C1	121.1(2)
C6	C1	C2	119.5(2)	C2	C3	C4	121.3(3)
C5	C4	C3	120.2(2)	C12	C13	C8	120.8(2)
C10	C9	C8	120.5(2)	C11	C10	C9	120.3(2)
C19	C20	Li30	85.82(17)	C16	C15	C14	121.9(2)
C9	C8	P7	118.65(18)	C18	C17	C16	121.3(2)
C9	C8	C13	118.6(2)	C20	Li30	P7	75.81(13)
C13	C8	P7	122.78(17)	C20	Li30	C19	33.65(10)
C25	N26	Li30	104.5(2)	N26	Li30	P7	113.45(18)
C25	N26	C27	109.4(3)	N26	Li30	C20	128.4(2)
C25	N26	C28	114.5(3)	N26	Li30	C19	102.08(18)
C27	N26	Li30	104.6(2)	N23	Li30	P7	127.79(19)
C28	N26	Li30	119.2(2)	N23	Li30	C20	128.1(2)
C28	N26	C27	104.2(3)	N23	Li30	N26	87.74(17)
C24	N23	Li30	103.2(2)	N23	Li30	C19	160.3(2)
C22	N23	Li30	107.5(2)	C19	Li30	P7	63.88(11)
C22	N23	C24	106.7(3)	C10	C11	C12	119.8(2)
C29	N23	Li30	116.1(2)	C19	C14	P7	115.42(17)
C29	N23	C24	109.3(3)	C15	C14	P7	123.07(18)
C29	N23	C22	113.2(3)	C15	C14	C19	120.7(2)
C20	C19	C18	122.1(2)	C4	C5	C6	119.3(3)
C20	C19	Li30	60.53(15)	C15	C16	C17	118.4(2)
C20	C19	C14	122.9(2)	C25	C24	N23	115.3(3)
C18	C19	Li30	124.06(18)	N26	C25	C24	114.8(3)

Table S 13. Hydrogen Atom Coordinates (Å×10^4) and Isotropic Displacement Parameters (Å^2×10^3) for **2-Li**.

Atom	x	y	z	U(eq)
H4	4203	1253	12207	46
H9	-589	1581	6490	30
H20A	5360(30)	1397(17)	7170(30)	34
H20B	4370(30)	1604(18)	7930(30)	34
H12	-1240	-218	8923	30
H18	5119	209	6167	28
H6	3603	293	9246	35
H3	2836	2192	11459	42
H13	784	186	8978	28
H10	-2614	1185	6454	35
H15	1229	-236	6916	34
H17	4048	-843	5563	33
H11	-2946	293	7675	32
H21A	1778	2683	8846	59
H21B	1442	2780	9990	59
H21C	665	2232	9118	59
H5	4546	283	11114	44
H16	2093	-1088	5934	39

H24A 1917 3365 4195 69
H24B 3115 2895 4176 69
H25A 726 2385 4161 65
H25B 1503 2241 3277 65
H27A 2673 1204 3328 84
H27B 3324 851 4467 84
H27C 3682 1652 4184 84
H28A 318 1180 4659 87
H28B 1383 583 4801 87
H28C 776 904 3630 87
H22A 1485 3759 5668 105
H22B 2004 3415 6840 105
H22C 1035 2976 5937 105
H29A 4585 3144 5729 104
H29B 4175 3458 6760 104
H29C 3792 3871 5627 104

Crystal Data for $C_{26}H_{34}LiN_2P$ (M =412.46 g/mol): monoclinic, space group P2$_1$/n (no. 14), a = 10.90505(11) Å, b = 18.17218(18) Å, c = 12.49103(13) Å, β = 103.7696(10)°, V = 2404.19(4) Å^3, Z = 4, T = 100.0(3) K, μ(CuKα) = 1.097 mm^{-1}, Dcalc = 1.140 g/cm^3, 8793 reflections measured (8.764° ≤ 2Θ ≤ 145.202°), 4677 unique (R_{int} = 0.0109, R_{sigma} = 0.0153) which were used in all calculations. The final R_1 was 0.0685 (I > 2σ(I)) and wR_2 was 0.1851 (all data).

Refinement model description

Number of restraints - 0, number of constraints - unknown.

Details:

1. Fixed Uiso
At 1.2 times of:
All C(H) groups, {H24A,H24B} of C24, {H25A,H25B} of C25
At 1.5 times of:
All C(H,H,H) groups, {H20A,H20B} of C20
2.a Secondary CH2 refined with riding coordinates:
C24(H24A,H24B), C25(H25A,H25B)
2.b Aromatic/amide H refined with riding coordinates:
C4(H4), C9(H9), C12(H12), C18(H18), C6(H6), C3(H3), C13(H13), C10(H10), C15(H15), C17(H17), C11(H11), C5(H5), C16(H16)
2.c Idealised Me refined as rotating group:
C21(H21A,H21B,H21C), C27(H27A,H27B,H27C), C28(H28A,H28B,H28C), C22(H22A,H22B, H22C), C29(H29A,H29B,H29C)

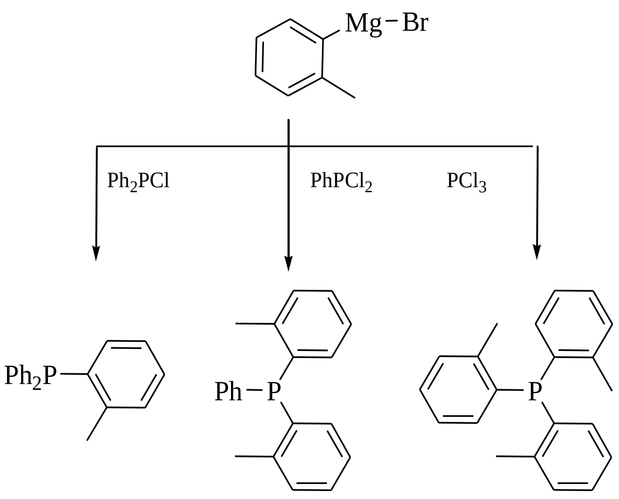

Ph2P(*o*-tol) PhP(*o*-tol)$_2$ P(*o*-tol)$_3$

Scheme S1. Synthesis of benzylphosphines ligands **1**, **2** and **3**.

4. DFT computations for **1a-Li**.

4.1 Cartesian coordinates

15	-0.767531001	-0.186762034	-0.211395927
6	0.047497975	2.083229169	3.114919325
1	-0.155486079	3.085122237	3.477974357
6	-0.280896983	0.344032025	1.455019202
6	-1.528860114	1.307938053	-0.954943982
7	3.161594271	0.615218178	0.121095098
6	-0.523289051	1.628805112	1.941516237
1	-1.159831125	2.301551138	1.370454196
6	1.138855142	-0.054152951	3.370020346
1	1.782146216	-0.716706977	3.947285392
6	-2.771636090	-1.889623243	-1.113166995
1	-2.311150059	-1.727476211	-2.087039070
6	3.770125316	0.587240196	-1.205843003
1	4.730165370	1.136772280	-1.214968003
1	3.099647243	1.123665215	-1.888980054
6	-4.471358176	-2.910847384	0.236447107
1	-5.343720233	-3.551107468	0.325490114
6	-2.836066230	1.713213032	-0.699257965
1	-3.463006256	1.116945961	-0.040569914
6	2.689549184	1.958852263	0.436581122
1	3.518073218	2.688577350	0.422282121
1	2.223651146	1.969013245	1.426007197
1	1.933394112	2.269991256	-0.290492934
7	2.765825327	-1.618835010	-1.683951038
6	-3.887609142	-2.700913344	-1.004705990
1	-4.303168157	-3.174543397	-1.889311054
6	-2.822139105	-1.485820213	1.250511182
1	-2.400116095	-1.006721160	2.130953252
6	-3.934537163	-2.307091317	1.361690195

1	-4.385291190	-2.474285345	2.335461266
6	-2.235833071	-1.265322171	0.010596090
6	0.847008190	-1.882180100	1.724540219
1	0.076127150	-2.428020172	1.179990180
1	1.410780254	-2.512910130	2.411101275
6	0.546907116	-0.576816013	2.184732255
6	0.894855074	1.219757136	3.817792380
1	1.359985096	1.556414183	4.741613453
6	1.924227249	-1.321775017	-2.835852130
1	1.650335186	-0.263506948	-2.855358127
1	0.994956201	-1.894310097	-2.770617122
1	2.434618298	-1.573852018	-3.782005202
6	4.094286355	0.175820180	1.152165178
1	4.402047419	-0.861190887	0.988715164
1	3.598344316	0.225739165	2.125802251
1	4.997169403	0.811504260	1.176656178
6	-2.540115281	3.643035192	-2.100865069
1	-2.935274349	4.551465245	-2.545196107
6	3.995498387	-0.825358898	-1.705701043
1	4.431423418	-0.797091880	-2.720230119
1	4.732157463	-1.327435911	-1.069288993
6	-3.337855312	2.871996100	-1.268794007
1	-4.358159405	3.178401084	-1.058377993
6	-1.239607167	3.247590210	-2.368395094
1	-0.613046144	3.843095281	-3.025618140
6	-0.743786088	2.083760140	-1.802704048
1	0.273188005	1.764215156	-2.028212065
6	3.063763400	-3.043123106	-1.647484037
1	2.130324351	-3.610984186	-1.603926034
1	3.644151455	-3.280395104	-0.751000970
1	3.632062459	-3.367378111	-2.536789105
3	1.746874219	-0.947092997	0.038740092

5. Polymerization reactions

5.1 NMR spectra of selected polymers before purification to determine conversion values

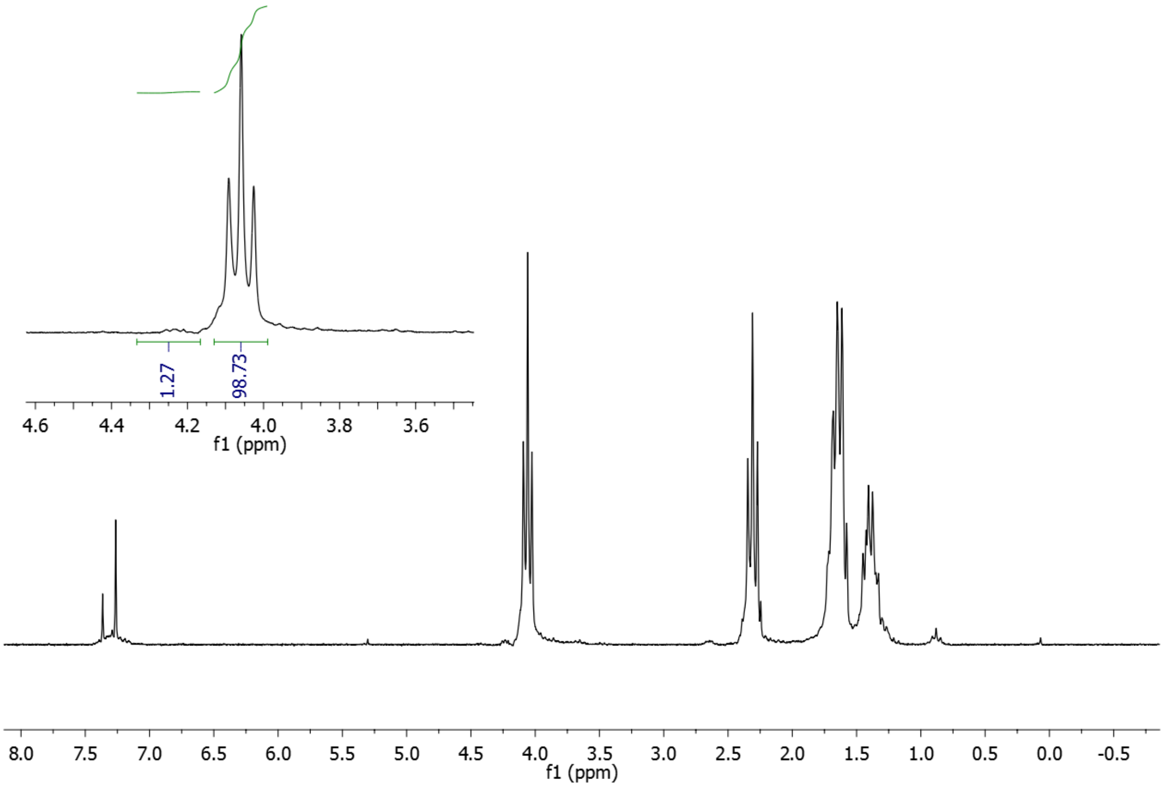

Figure S25. ^{1}H NMR spectrum of PCL obtained with catalyst **1-Li** (Table 1, entry 1).

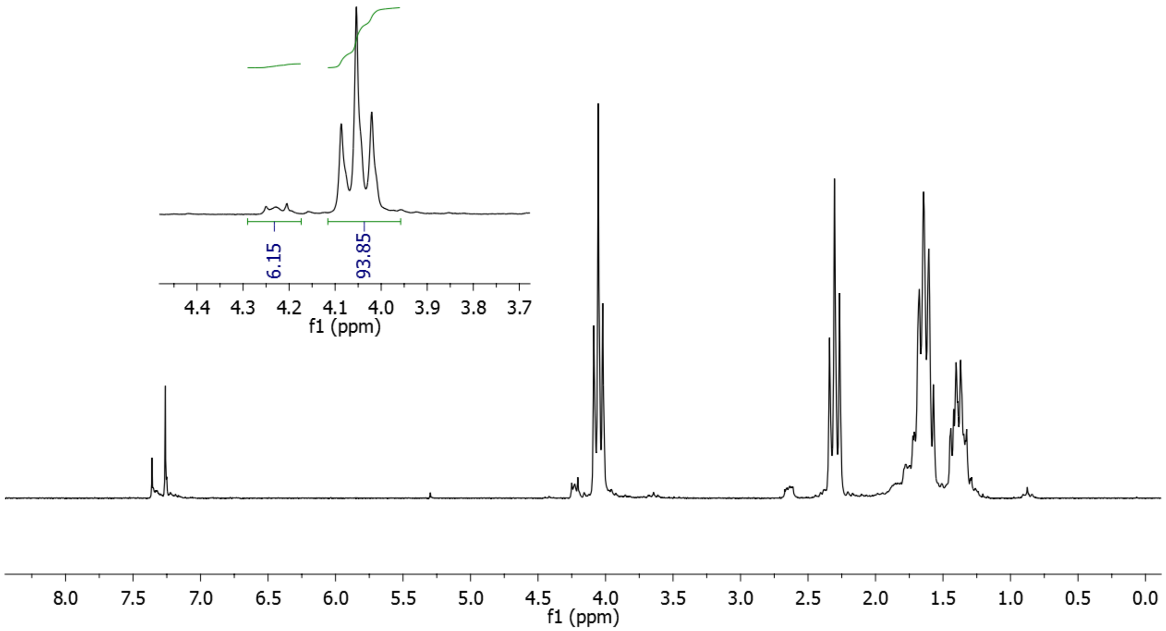

Figure S26. ^{1}H NMR spectrum of PCL obtained with catalyst **2-Li** (Table 1, entry 2).

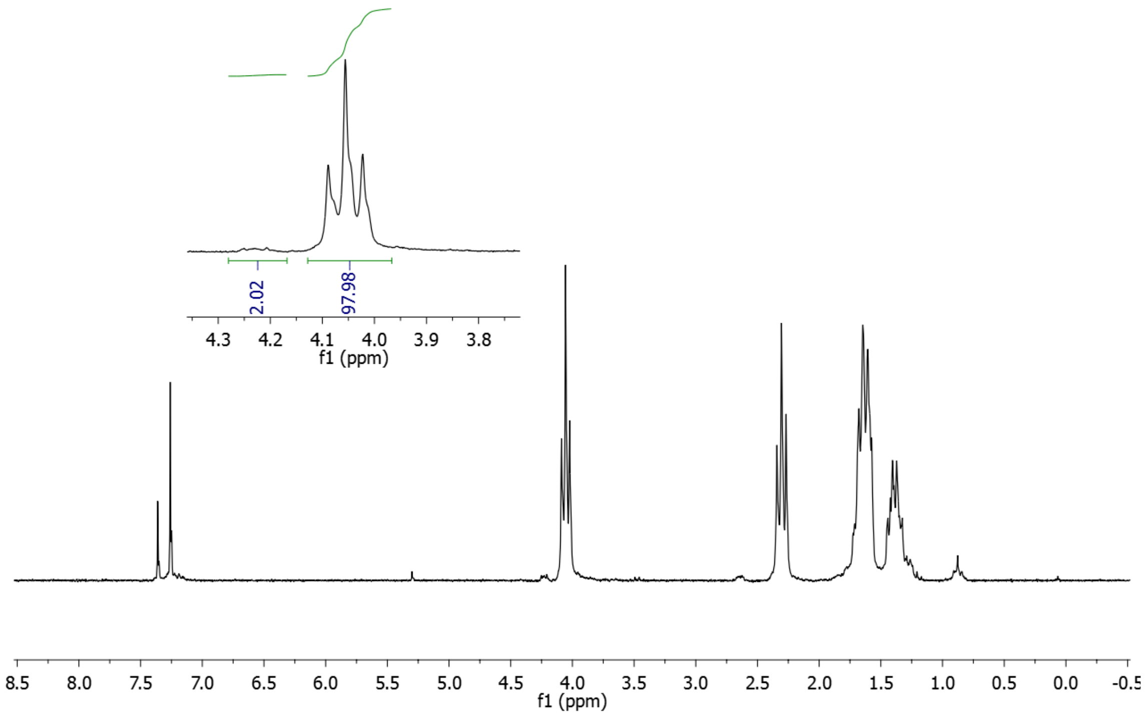

Figure S27. ^{1}H NMR spectrum of PCL obtained with catalyst **2-Li$_2$** (Table 1, entry 3).

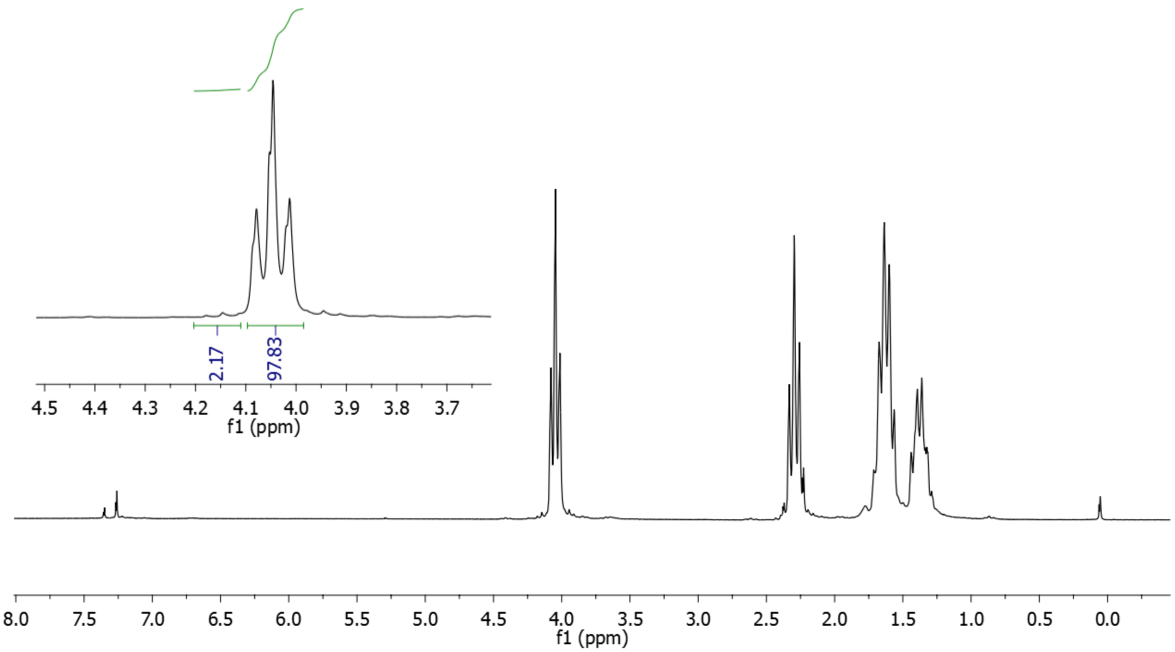

Figure S28. ^{1}H NMR spectrum of PCL obtained with catalyst **3-Li₃** (Table 1, entry 4)

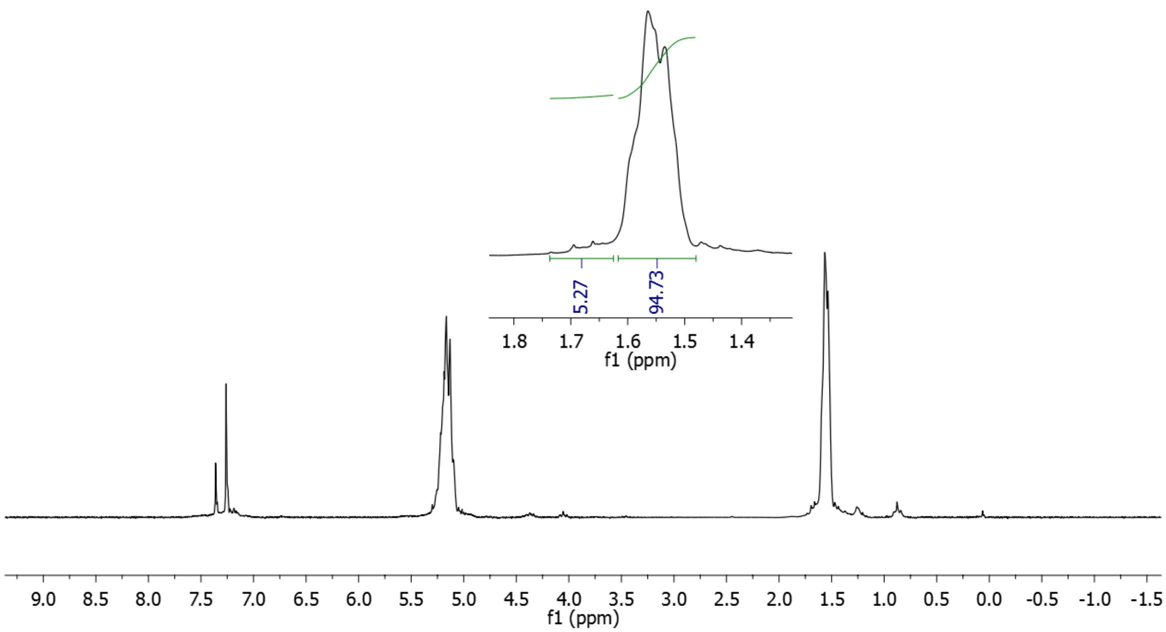

Figure S29. ^{1}H NMR spectrum of PLA obtained with catalyst **2-Li** (200 MHz, Table 2, entry 2).

5.2 NMR spectra of selected polymers following purification

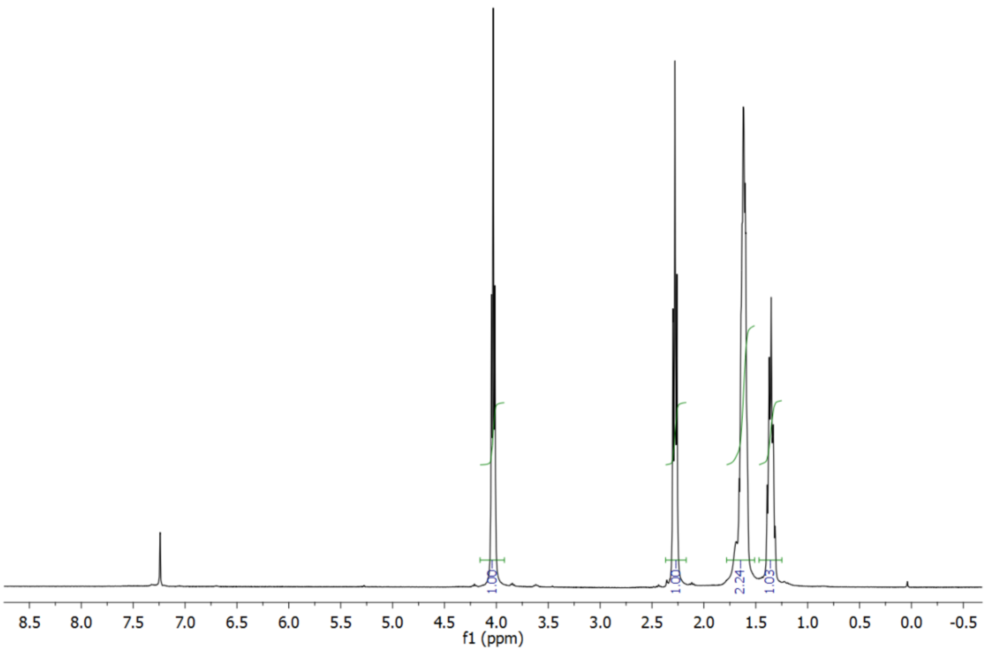

Figure S30. ^{1}H NMR spectrum of PCL obtained with catalyst **2-Li** (Table 1, entry 2).

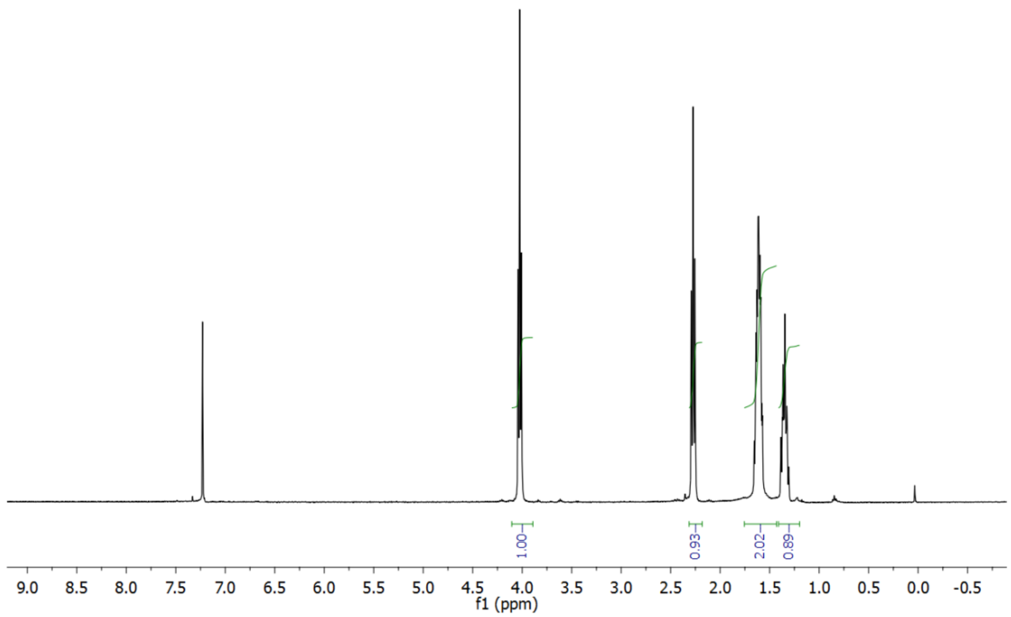

Figure S31. ^{1}H NMR spectrum of PCL obtained with catalyst **3-Li$_3$** (Table 1, entry 4).

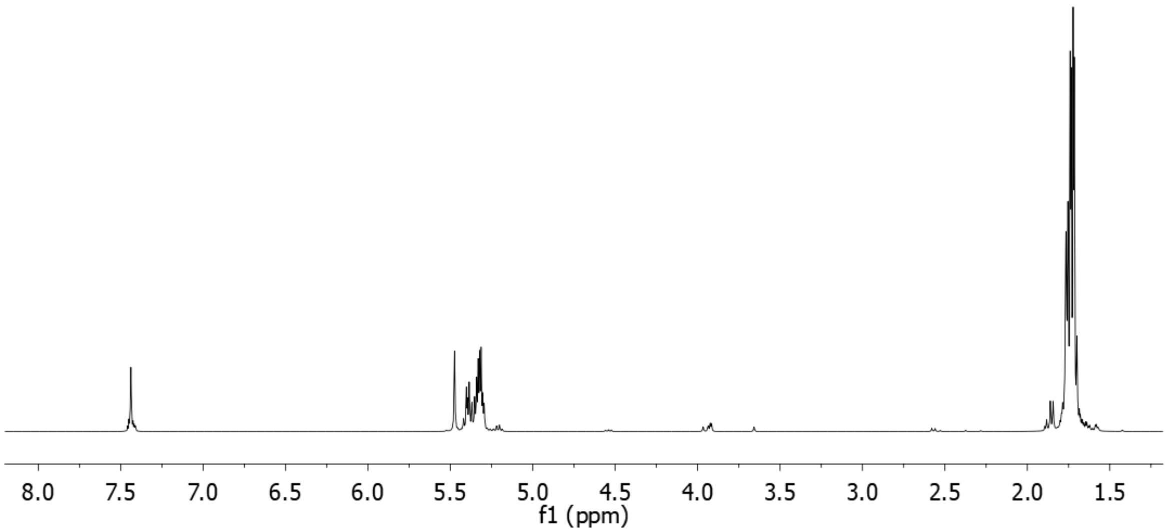

Figure S32. ^{1}H NMR of PLA obtained with catalyst **2-Li** (500 MHz, Table 2, entry 2).

5.3 Calculation procedure for determining the isotactic probability of the polylactides from the methine region of their ^{1}H NMR spectra giving rise to the values shown in Table 2.

The calculation procedure for the determination of the isotactic probability of the polymers was based on the methods reported in the references below:

- Coudane, J., et al. (1997). "More about the stereodependence of DD and LL pair linkages during the ring-opening polymerization of racemic lactide." Journal of Polymer Science Part A: Polymer Chemistry **35**(9): 1651-1658.

- Chamberlain, B. M., et al. (2001). "Polymerization of Lactide with Zinc and Magnesium β-Diiminate Complexes: Stereocontrol and Mechanism." Journal of the American Chemical Society **123**(14): 3229-3238.

- Xiong, J., et al. (2015). "Iso-Selective Ring-Opening Polymerization of rac-Lactide Catalyzed by Crown Ether Complexes of Sodium and Potassium Naphthalenolates." Inorganic Chemistry **54**(4): 1737-1743.

Herein, we show a representative example of the methine region of one of our polymers and of the calculations giving rise to the results presented in Table 2 of the manuscript.

Tetrad	Area	Normalized area
rmr	45095,093	0,13575758
rmm	40535,392	0,12203072
mmr	70963,792	0,21363461
mmm	72095,727	0,21704227
mrm	103483,658	0,31153481

$P_r = (2(0.1357))^{1/2}$

$P_r = 0.52$

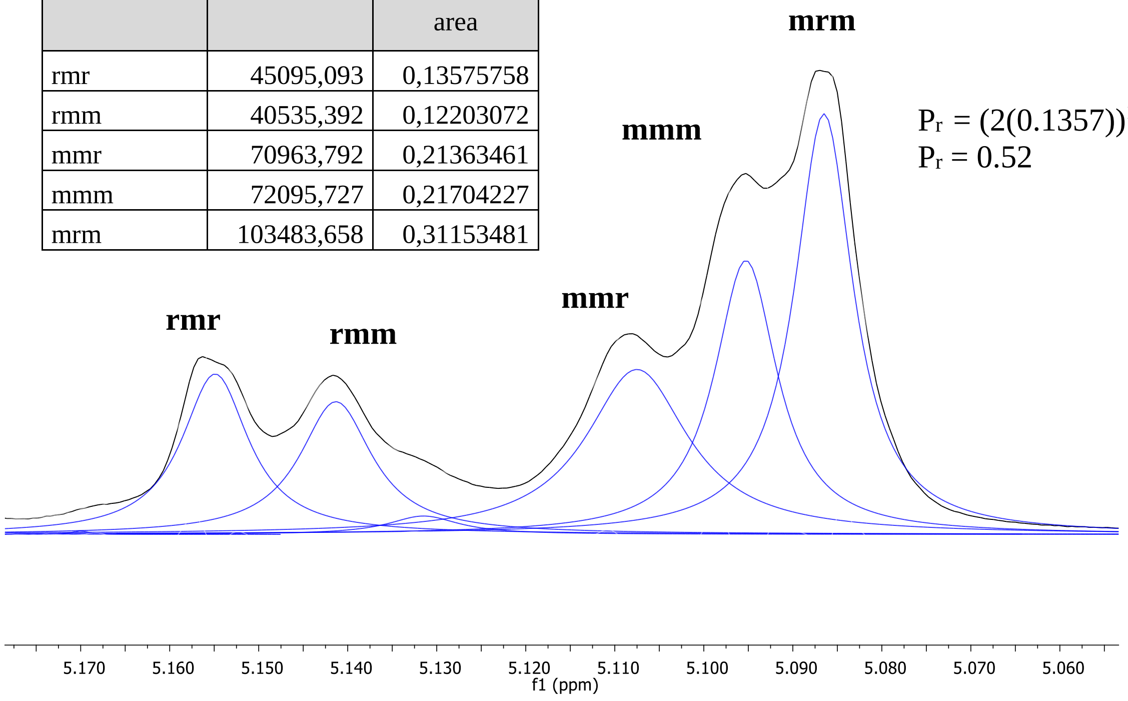

Figure S 33. Deconvolution of the methine region of the homonuclear-decoupled ^{1}H NMR spectrum of PLA using **2-Li** (Table 2, entry 2).

5.4 Yields of isolated polymers

Table S 14. Polymerization of ε-CL by lithium complexes at 25°C including observed conversion and yields of isolated polymers. Modified from Table 1 in main text. Cat = catalyst.

Entry	Cat	$[\varepsilon\text{-CL}]_0/[\text{Cat}]_0$	Conversion [a] (%)	Yield [b] (%)
**1**	**1-Li**	100	99	77
**2**	**2-Li**	100	94	80
**3**	**2-Li$_2$**	100	98	90
**4**	**3-Li$_3$**	100	98	92

[a] Conversion was determined by ^{1}H NMR of $CDCl_3$ solutions of the polymers formed after the reaction times indicated in Table 1 and according to the description in the Experimental Part of the main text. [b] The yield was determined from gravimetric analysis of the isolated polymer after purification as described in the Experimental Part.

Table S 15. Polymerization of *rac*-LA by lithium complexes at 140°C including yields of isolated polymers. Modified from Table 2 in main text. Cat= catalyst.

Entry	Cat.	[*rac*-LA]$_0$/[Cat]$_0$	Conversion [a] (%)	Yield[b] (%)
**1**	**1-Li**	100	93	92
**2**	**2-Li**	100	95	89
**3**	**2-Li$_2$**	100	97	90
**4**	**3-Li$_3$**	100	98	95

[a] Conversion was determined by ^{1}H NMR of $CDCl_3$ solutions of the polymers formed after the reaction times indicated in Table 2 and according to the description in the Experimental Part of the main text. [b] The yield was determined from gravimetric analysis of the isolated polymer after purification as described in the Experimental Part.
